# Obstructive Sleep Apnea Risk and Mental Health Conditions Among Older Canadian Adults in the Canadian Longitudinal Study on Aging

**DOI:** 10.1001/jamanetworkopen.2025.49137

**Published:** 2025-12-26

**Authors:** Tetyana Kendzerska, Ranjeeta Mallick, Wenshan Li, Rébecca Robillard, Vanessa Taler, Colleen Webber, Mouaz Saymeh, Thien Thanh Dang-Vu, Peter Tanuseputro, Jess G. Fiedorowicz

**Affiliations:** 1 Inflammation and Chronic Disease Program, Ottawa Hospital Research Institute, Ottawa, Ontario, Canada; 2 Department of Medicine, Faculty of Medicine, University of Ottawa, Ottawa, Ontario, Canada; 3 School of Epidemiology and Public Health, University of Ottawa, Ottawa, Ontario, Canada; 4 Ottawa Methods Centre, Ottawa Hospital Research Institute, Ottawa, Ontario, Canada; 5 Ottawa Hospital Research Institute, Ottawa, Ontario, Canada; 6 School of Psychology, University of Ottawa, Ottawa, Ontario, Canada; 7 Sleep Research Unit, Royal Ottawa Institute of Mental Health Research, Ottawa, Ontario, Canada; 8 Bruyere Health Research Institute, Ottawa, Ontario, Canada; 9 Department of Health, Kinesiology, and Applied Physiology, Concordia University, Montréal, Québec, Canada; 10 Centre de recherche de l’Institut universitaire de gériatrie de Montréal (CRIUGM), Centre intégré universitaire de soins et services sociaux (CIUSSS) du Centre-Sud-de-l’île-de-Montréal, Montréal, Québec, Canada; 11 Department of Family Medicine and Primary Care, Faculty of Medicine, The University of Hong Kong, Hong Kong, Hong Kong, SAR of China; 12 Neuroscience Program, Ottawa Hospital Research Institute, Ottawa, Ontario, Canada; 13 Department of Psychiatry, University of Ottawa, Ottawa, Ontario, Canada

## Abstract

**Importance:**

Despite plausible mechanisms linking obstructive sleep apnea (OSA) and mental health conditions, prospective studies from representative samples are needed to estimate temporal associations between OSA and mental health conditions during aging.

**Objective:**

To evaluate whether high risk of OSA is associated with increased odds of concurrent and future mental health conditions among middle-aged and older adults.

**Design, Setting, and Participants:**

This cohort study is a secondary analysis of the Canadian Longitudinal Study on Aging (CLSA) and used data from respondents of the CLSA Baseline Comprehensive Cohort (2011-2015) and Follow-up 1 (2015-2018) who were aged 45 to 85 years at baseline. Statistical analysis was performed October 2024. The CLSA is a national community-based prospective cohort study collecting data on the biological, medical, cognitive, psychological, social, lifestyle, and economic aspects of aging.

**Exposure:**

Individuals with a score greater or equal to 2 on the STOP (snoring, daytime somnolence, witnessed apnea during sleep, or hypertension) questionnaire were considered at high risk of OSA.

**Main Outcome and Measures:**

A composite poor mental health outcome was computed as a binary variable, defined by the presence of any of the following: (1) Center for Epidemiologic Studies Short Depression Scale score of 10 or more, (2) Kessler Psychological Distress Scale score of 20 or more, (3) self-reported physician-diagnosed mental health condition, or (4) self-reported antidepressant use. Multivariate conventional and mixed logistic regressions were used to examine associations.

**Results:**

The study included 30 097 individuals at baseline (median age, 62 years [IQR, 54-71 years]; 50.9% women) and 27 765 individuals at follow-up (median age, 65 years [IQR, 57-73 years]; 50.9% women), with a median follow-up of 2.9 years (IQR, 2.8-3.1 years). A total of 7066 of 30 097 individuals (23.5%) at baseline and 7493 of 27 765 individuals (27.0%) at follow-up were at high risk of OSA. The composite mental health outcome was identified in 10 334 of 30 097 individuals (34.3%) at baseline and 8851 of 27 765 individuals (31.9%) at follow-up. In adjusted models, high risk of OSA was associated with an approximately 40% higher odds of the composite outcome concurrently at baseline (odds ratio [OR], 1.39; 95% CI, 1.28-1.50) and at follow-up (OR, 1.40; 95% CI, 1.30-1.50). In a repeated-measures analysis, OSA risk remained associated with a 44% higher odds (OR, 1.44; 95% CI, 1.34-1.53) of the composite outcome.

**Conclusions and Relevance:**

In this national longitudinal cohort study, middle-aged and older adults at high risk of OSA had consistently worse mental health outcomes. These findings bridge knowledge gaps on the association between OSA and mental health, highlighting the need for integrated screening and intervention strategies.

## Key Points

**Question:**

Is high risk of obstructive sleep apnea (OSA) associated with increased odds of concurrent and future mental health conditions among middle-aged and older adults?

**Findings:**

In this national cohort study of 30 097 individuals, those at high risk of OSA had approximately 40% higher odds of mental health conditions at both baseline and follow-up. Over time, high risk of OSA remained associated with a 44% increased odds of reporting new mental health conditions.

**Meaning:**

These findings bridge knowledge gaps on the association between OSA and mental health during aging, highlighting the need for integrated screening and intervention strategies.

## Teaser

This cohort study evaluates whether high risk of obstructive sleep apnea is associated with increased odds of concurrent and future mental health conditions among middle-aged and older adults.

## Introduction

Mental health conditions are among the leading contributors to global disease burden, with anxiety and depressive disorders being the most common.^1^ Individuals with mental health conditions face higher risks of cardiometabolic diseases, unemployment, homelessness, disability, and hospitalizations.^2^ Mental disorders cost $1 trillion annually globally in lost productivity.^3^ Identifying factors associated with mental health outcomes remains an important public health goal.

Obstructive sleep apnea (OSA) is a prevalent yet underdiagnosed condition, characterized by repeated upper airway narrowing during sleep, resulting in sleep fragmentation, sympathetic activation, and intermittent hypoxemia.^4^ OSA affects an estimated 936 million adults aged 30 to 69 globally,^5^ with up to 90% of cases undetected.^6,7^ It has been linked with cardiometabolic diseases and greater health care use^8,9,10,11,12,13,14^ and is associated with traffic and occupational accidents and reduced productivity.^15,16^ OSA is a treatable condition, with evidence-based cost-effective therapies^17^ that can improve symptoms and reduce long-term health risks.^8^

Through hypoxemia and sleep fragmentation, untreated OSA may be associated with the development and progression of mental health conditions.^18^ In turn, mental health conditions may be associated with increases in OSA risk via weight gain and altered upper airway muscle tone due to autonomic imbalance, neurotransmitter dysregulation, and neuromuscular impairment.^19^ Despite these plausible mechanisms, existing research is limited by small sample sizes, single-center studies, and inadequate adjustment for confounders (eg, no adjustment or adjustment for demographic characteristics only),^11,18,20^ limiting conclusions about the cross-sectional and longitudinal associations.^21^ OSA is also underdiagnosed or untreated among individuals with mental health conditions,^22,23^ suggesting that the unmet burden of treatable OSA may further worsen mental health outcomes. There is a need for prospective studies in representative samples to estimate temporal associations.

To address this need, we conducted a secondary analysis of the prospective Canadian Longitudinal Study on Aging (CLSA). Our first objective was to evaluate whether high risk of OSA is associated with increased odds of concurrent and future mental health conditions among middle-aged and older adults. Our second, exploratory objective was to identify individual characteristics (sociodemographic and lifestyle measures and comorbid sleep and medical conditions) associated with new mental health conditions among individuals at high risk of OSA. We hypothesize that high risk of OSA is independently associated with an increased risk of mental health conditions, both concurrently and longitudinally, and that distinct risk profiles for new mental health conditions can be identified among individuals at high risk of OSA.

## Methods

### Study Design, Population, and Data Sources

We used data from 45- to 85-year-old (age at baseline) respondents of the CLSA national community-based prospective cohort study that has been collecting data on the biological, medical, cognitive, psychological, social, lifestyle, and economic aspects of middle-aged and older adults without cognitive impairment at baseline.^24^ The CLSA study design and recruitment process have been published elsewhere.^25,26^ Individuals living on federal First Nations reserves, full-time members of the Canadian Armed Forces, residents of institutions, and those unable to respond in English or French or with cognitive impairment were not recruited by CLSA^25,26^ (eMethods in Supplement 1). Ethical approval for the CLSA was obtained from research ethics boards at all participating institutions. All participants provided written informed consent. The analyses presented in this article were conducted under a CLSA data application number (23CA001) and were approved by the Hamilton Integrated Research Ethic Board and the Ottawa Hospital Research Institute. This study followed the Strengthening the Reporting of Observational Studies in Epidemiology (STROBE) reporting guideline for cohort studies.^27^

For this study, we used data from the Baseline Comprehensive Cohort (2011-2015; N = 30 097), for which data were collected by face-to-face interviews,^25^ and from Follow-up 1 (2015-2018; N = 27 765). More details on definitions of variables are presented in eTable 1 in Supplement 1 and at the study website.^24^

### Exposures: Self-Reported High Risk of OSA

The primary exposure, high risk of OSA, was defined using the validated STOP questionnaire,^28^ which classifies high risk of OSA when at least 2 of the following are reported: snoring, daytime somnolence, witnessed apnea during sleep, and/or hypertension. Meta-analyses and systematic reviews show that the STOP questionnaire (score ≥2) yields a sensitivity of 87% to 90% and a specificity of 29% to 42% for any OSA (apnea-hypopnea index [AHI] ≥5), with a negative predictive value (NPV) typically above 80% for moderate to severe OSA and a positive predictive value (PPV) below 50% in community samples.^29,30,31,32,33^

Hypertension was defined as systolic blood pressure of at least 140 mm Hg or diastolic blood pressure of at least 90 mm Hg (mean of 4 measures) or self-reported diagnosis of hypertension or antihypertensive medication use.

The secondary exposure was defined by a single question: “Has anyone ever observed you stop breathing in your sleep?” (yes or no). In contrast, witnessed apnea alone has a sensitivity ranging from 20% to 40% and a specificity of 80% to 95%, with a PPV often exceeding 60% and an NPV lower than the STOP questionnaire, due to the lower prevalence of witnessed events in the general population.^30,34^

### Mental Health–Related Outcomes

The primary mental health–related outcome was a composite of poor mental health as a binary variable, defined by the presence of any of the following: (1) a 10-item Center for Epidemiologic Studies Short Depression Scale (CES-D-10)^35,36^ score of 10 or more, (2) a 10-item Kessler Psychological Distress Scale (K10)^37^ score of 20 or more, (3) self-reported physician-diagnosed mental health condition (“Has a doctor ever told you . . .?”), or (4) self-reported antidepressant use (“Are you currently taking medications for depression?”).

The CES-D-10 is a 10-item scale, with higher scores indicating greater depressive symptoms. A cutoff of 10 or more identifies clinically relevant depression symptoms (sensitivity, 97%; specificity, 84%; and PPV, 85% for major depressive disorder).^38,39^ The K10 is a 10-item scale assessing symptoms of psychological distress,^40^ with high internal consistency (α = 0.88) and convergent validity (α = 0.84),^37,40^ and a recommended cutoff of 20 or more.^37^ Mental health conditions were based on self-reported physician diagnoses of an anxiety disorder “such as a phobia, obsessive-compulsive disorder, or a panic disorder,” a mood disorder “such as depression (including manic depression), bipolar disorder, mania, or dysthymia,” or “clinical depression.”

The secondary mental health–related outcomes were physician-diagnosed anxiety disorder, mood disorder, and clinical depression, considered separately. Although clinical depression falls under mood disorder, the survey asked about these separately, and participants likely viewed them as distinct. Therefore, we report them as separate outcomes.

### Potential Covariates and Risk Factors

Potential covariates were selected based on the literature review^11,41,42^ and expert opinion. Self-reported sociodemographic and lifestyle measures^43^ included age, sex, race and ethnicity (Arab only, Black only, Chinese only, Filipino only, Japanese only, Korean only or West Asian only [combined due to low numbers], Latin American only, South Asian only, Southeast Asian only, White only, other racial or ethnic origin only [not specified], and multiple racial or ethnic origins), urban and marital status, dwelling type, household income, educational level, self-rated general health, satisfaction with life (by Satisfaction with Life Scale),^44^ functional social support, smoking status, alcohol consumption, and physical activity. Body mass index (BMI; calculated as weight in kilograms divided by height in meters squared) was based on height and weight measured at the interview. The CLSA collects information on race and ethnicity as part of its core sociodemographic profile to understand diversity in aging experiences across Canada.

Measures of sleep-related symptoms and other sleep disorders aside from OSA risk^43,45^ included dissatisfaction with sleep pattern, number of hours of daily sleep, insomnia symptoms, acting out on dreams while asleep, and restless leg syndrome (adapted from validated sleep questionnaires).^46,47^ Medical conditions included self-reported number of medications prescribed and a physician-diagnosed (“Has a doctor ever told you . . .?”) chronic condition of interest, such as cardiovascular conditions, diabetes, hypothyroidism, respiratory conditions or problems, cancer, traumatic brain injury, and intensity of pain or discomfort. Self-reported and clinician-diagnosed chronic conditions demonstrated high test-retest reliability in population-based surveys.^25,48^

### Variable Selection Process for the Final Models

From the set of 28 variables described, we implemented several steps to select a joint core set of variables for the final models, addressing each objective separately for the baseline and follow-up cohorts (eMethods in Supplement 1). First, we removed variables with more than 10% missing values to preserve the sample size. Then, we excluded collinear variables based on a variable clustering algorithm. Finally, we used the step-down procedure described by Ambler et al,^49^ where variables are sequentially deleted to obtain the lowest, stable Akaike information criterion value, selecting the final models, which included 19 variables for baseline and 17 variables for follow-up (Table 1). All statistical models were additionally adjusted for the year of data collection.

**Table 1.** Population Characteristics of Variables Included in the Statistical Model at Baseline and Follow-Up

Variable	Baseline, No. (%)			*P* value	Follow-up, No. (%)			*P* value
	Total (N = 30 097)	High risk of OSA at baseline^a^			Total (N = 27 765)	High risk of OSA at follow-up^b^		
		Yes (n = 7066)	No (n = 20 205)			Yes (n = 7493)	No (n = 17 528)	
Start date year								
2011-2012	3803 (12.6)	842 (11.9)	2572 (12.7)	<.001	NA	NA	NA	.001
2013	11 476 (38.1)	2516 (35.6)	7803 (38.6)		NA	NA	NA	
2014	10 363 (34.4)	2457 (34.8)	6993 (34.6)		NA	NA	NA	
2015	4455 (14.8)	1251 (17.7)	2837 (14.0)		4328 (15.6)	1159 (15.5)	2719 (15.5)	
2016	NA	NA	NA		10 103 (36.4)	2616 (34.9)	6548 (37.4)	
2017	NA	NA	NA		10 656 (38.4)	2963 (39.5)	6644 (37.9)	
2018	NA	NA	NA		2678 (9.7)	755 (10.1)	1617 (9.2)	
Missing^c^	NA	2826	2826		NA	2744	2744	
Sociodemographic and lifestyle measures								
Age groups, y								
45-54	7595 (25.2)	1439 (20.4)	5646 (27.9)	<.001	4394 (15.8)	988 (13.2)	3149 (18.0)	<.001
55-64	9856 (32.8)	2486 (35.2)	6595 (32.6)		9173 (33.0)	2522 (33.7)	6014 (34.3)	
65-74	7362 (24.5)	1940 (27.5)	4675 (23.1)		8243 (29.7)	2506 (33.4)	4917 (28.1)	
≥75	5284 (17.6)	1201 (17.0)	3289 (16.3)		5955 (21.5)	1477 (19.7)	3448 (19.7)	
Sex								
Female	15 320 (50.9)	2688 (38.0)	11 043 (54.7)	<.001	14 133 (50.9)	2889 (38.6)	9695 (55.3)	<.001
Male	14 777 (49.1)	4378 (62.0)	9162 (45.3)		13 632 (49.1)	4604 (61.4)	7833 (44.7)	
Dwelling type								
House	24 004 (79.8)	5634 (79.8)	16 462 (81.5)	<.001	21 671 (78.1)	5878 (78.5)	14 001 (79.9)	.01
Apartment or condominium	5810 (19.3)	1347 (19.1)	3590 (17.8)		5474 (19.7)	1456 (19.4)	3201 (18.3)	
Other	273 (0.9)	Not reported^d^	Not reported^d^		619 (2.2)	46 (0.6)	89 (0.5)	
Household income								
<$50 000	7926 (26.3)	2004 (30.2)	4682 (24.7)	<.001	6879 (24.8)	1942 (27.6)	3847 (23.3)	<.001
≥$50 000 to <$100 000	9907 (32.9)	2348 (35.4)	6783 (35.8)		9406 (33.9)	2603 (36.9)	5964 (36.2)	
≥$100 000 to <$150 000	5524 (18.4)	1282 (19.3)	3897 (20.6)		5124 (18.5)	1390 (19.7)	3411 (20.7)	
≥$150 000	4799 (16.0)	1001 (15.1)	3584 (18.9)		4585 (16.5)	1112 (15.8)	3258 (19.8)	
Life satisfaction								
Dissatisfied or neutral	4126 (13.7)	1225 (17.5)	2308 (11.6)	<.001	3470 (12.5)	1176 (15.9)	1782 (10.3)	<.001
Slightly satisfied	4471 (14.9)	1205 (17.2)	2803 (14.0)		3441 (12.4)	1047 (14.2)	1986 (11.5)	
Satisfied	9221 (30.6)	2175 (31.1)	6220 (31.1)		8222 (29.6)	2241 (30.3)	5163 (29.8)	
Extremely satisfied	11 925 (39.6)	2395 (34.2)	8652 (43.3)		12 276 (44.2)	2931 (39.6)	8407 (48.5)	
Self-rated general health								
Excellent	5995 (19.9)	765 (10.8)	4834 (23.9)	<.001	5071 (18.3)	911 (12.2)	3791 (21.7)	<.001
Very good	12 420 (41.3)	2518 (35.7)	8869 (43.9)		11 460 (41.3)	2704 (36.2)	7798 (44.5)	
Good	8877 (29.5)	2612 (37.0)	5260 (26.1)		8199 (29.5)	2661 (35.6)	4631 (26.4)	
Fair	2315 (7.7)	949 (13.4)	1046 (5.2)		2450 (8.8)	982 (13.1)	1082 (6.2)	
Poor	467 (1.6)	216 (3.1)	182 (0.9)		547 (2.0)	221 (3.0)	212 (1.2)	
Current smoker								
Yes	2576 (8.6)	642 (12.5)	1565 (11.7)	.16	1944 (7.0)	600 (8.0)	1088 (6.2)	<.001
No	17 918 (59.5)	4502 (87.5)	11 780 (88.3)		25 792 (92.9)	6887 (92.0)	16 421 (93.8)	
Ever alcohol consumption	29 383 (97.6)	6907 (97.8)	19 747 (97.7)	.95	27 033 (97.4)	7310 (97.6)	17 072 (97.4)	.46
Type of drinker (past 12 mo)								
Regular drinker (at least once a month)	22 239 (73.9)	5010 (72.6)	15 385 (77.9)	<.001	20 889 (75.2)	5522 (73.7)	13 588 (77.6)	<.001
Occasional drinker	3705 (12.3)	987 (14.3)	2277 (11.5)		3405 (12.3)	970 (13.0)	1945 (11.1)	
Did not drink in the last 12 mo	3427 (11.4)	907 (13.1)	2078 (10.5)		3450 (12.4)	996 (13.3)	1984 (11.3)	
BMI								
Underweight	217 (0.7)	13 (0.2)	180 (0.9)	<.001	210 (0.8)	24 (0.3)	167 (1.0)	<.001
Normal weight	8863 (29.5)	1004 (14.3)	7146 (35.5)		7936 (28.6)	1151 (15.6)	6261 (36.2)	
Overweight	12 088 (40.2)	2624 (37.4)	8309 (41.3)		10 738 (38.7)	2743 (37.3)	7184 (41.6)	
Obesity class I	5820 (19.3)	1929 (27.5)	3273 (16.3)		5162 (18.6)	2002 (27.2)	2658 (15.4)	
Obesity class II	1978 (6.6)	887 (12.7)	904 (4.5)		1777 (6.4)	904 (12.3)	713 (4.1)	
Obesity class III	995 (3.3)	557 (7.9)	329 (1.6)		922 (3.3)	538 (7.3)	301 (1.7)	
Medical conditions								
No. medications taken, median (IQR)	4 (2-7)	5 (2-8)	3 (1-6)	<.001	4 (2-7)	5 (2-8)	3 (1-6)	<.001
TBI	7288 (24.2)	2015 (28.5)	4641 (23.0)	<.001	6475 (23.3)	2135 (29.0)	3898 (22.6)	<.001
Free of pain and discomfort	18 130 (60.2)	3749 (53.7)	13 517 (67.0)	<.001	18 208 (65.6)	4444 (59.4)	12 435 (71.1)	<.001
Usual intensity of pain or discomfort								
Mild	4589 (15.3)	1243 (39.0)	3075 (46.8)	<.001	3864 (13.9)	1208 (40.1)	2391 (47.5)	<.001
Moderate	4901 (16.3)	1547 (48.6)	3007 (45.7)		4119 (14.8)	1439 (47.8)	2261 (44.9)	
Severe	972 (3.2)	394 (12.4)	496 (7.5)		862 (3.1)	366 (12.2)	385 (7.6)	
Diabetes	5310 (17.6)	2025 (28.7)	2674 (13.3)	<.001	5307 (19.1)	2227 (30.1)	2599 (15.0)	<.001
Hypertension	14 127 (46.9)	5777 (82.1)	6601 (32.9)	<.001	13 714 (49.4)	6273 (85.0)	6088 (35.6)	<.001
Respiratory problem	7990 (26.6)	2347 (33.5)	4787 (23.8)	<.001	7337 (26.4)	2548 (34.5)	4175 (24.2)	<.001
CVD or stroke	3301 (11.0)	1182 (16.9)	1667 (8.3)	<.001	3236 (11.7)	1293 (17.5)	1581 (9.3)	.10
Underactive thyroid gland	3962 (13.2)	949 (13.6)	2583 (12.9)	.16	3989 (14.4)	1065 14.5	2557 14.8	.48
Sleep symptoms and other sleep disorders aside from OSA								
No. of hours of sleep per night, median (IQR)	7 (6-8)	7 (6-8)	7 (6-8)	<.001	7 (6-8)	7 (6-8)	7 (6-8)	<.001
Insomnia	1299 (4.3)	564 (8.0)	608 (3.0)	<.001	992 (3.6)	458 (6.2)	473 (2.7)	<.001
Restless leg syndrome	7809 (26.0)	2272 (32.3)	4788 (23.8)	<.001	5032 (18.1)	1704 (22.9)	2920 (16.7)	<.001
Acts out dreams while asleep^e^	3328 (11.1)	1096 (15.7)	1921 (9.6)	<.001	2859 (10.3)	1121 (15.3)	1563 (91.0)	<.001

Abbreviations: BMI, body mass index (calculated as weight in kilograms divided by height in meters squared); CVD, cardiovascular disease; NA, not applicable; OSA, obstructive sleep apnea; TBI, traumatic brain injury.

^a^ Numbers do not total 30 097 because of missing data for OSA status. The final list of variables at baseline (N = 19) included age, sex, dwelling type, total household income, self-rated general health, satisfaction with life, alcohol consumption, BMI, self-reported hypertension, diabetes, respiratory problem, usual intensity of pain or discomfort, positive screen for TBI, underactive thyroid gland, the number of medications taken, self-reported acting out on dream, restless legs, number of sleep hours per night, insomnia with daytime impairment.

^b^ Numbers do not total 27 765 because of missing data for OSA status. The final list of variables at follow-up (N = 17) included age, sex, dwelling type, total household income, satisfaction with life, current smoker status, alcohol consumption, self-reported CVD and cerebrovascular conditions, diabetes, respiratory problem, usual intensity of pain or discomfort, positive screen for TBI, underactive thyroid gland, self-reported acting out on dream, restless legs, number of sleep hours per night, insomnia with daytime impairment.

^c^ The difference in the total and by OSA status is explained by the missing values by the OSA status. More details on missing values per each variable are presented in eTable 2 in Supplement 1.

^d^ Not reported to avoid cells with fewer than 5.

^e^ For example, punching, flailing arms in the air, and making running movements.

### Statistical Analysis

Statistical analysis was performed October 2024 using SAS, version 9.4 (SAS Institute Inc). Descriptive statistics, such as mean (SD) values, median (IQR) values, or frequency (%), were used to characterize the study populations in total and by the primary exposure (high risk of OSA). All *P* values were from 2-sided tests and results were deemed statistically significant at *P* < .05.

#### Primary Analyses

To address the first objective, our primary analyses involved a series of multivariable logistic regression analyses. A cross-sectional analysis was performed to investigate the association between high risk of OSA and concurrent composite outcome at baseline and follow-up, separately, using multivariate logistic regressions. A longitudinal analysis was performed among individuals without mental health conditions at baseline, to assess the association between high risk of OSA at baseline and new mental health conditions at follow-up, using multivariable logistic regressions. A repeated-measures analysis was performed for participants with data available at both baseline and follow-up; we used mixed-effects multivariable logistic regressions to examine associations between high risk of OSA and new mental health conditions, incorporating repeated observations per individual and accounting for within-individual correlation through random intercepts (eMethods in Supplement 1).

#### Secondary Analyses

The same analytic approaches described were applied for the secondary exposure (witnessed apnea) and outcomes (physician-diagnosed anxiety disorder, mood disorder, and clinical depression, considered separately). Multivariable mixed-effects linear regressions were used to assess the association between high risk of OSA risk and longitudinal CES-D-10 or K10 scores, considered separately as continuous variables. Finally, we conducted several sensitivity analyses, incorporating all variables considered for selection along with urban residence, racial and ethnic background, and interaction terms between sex, age, and the primary exposure regardless of missing values and collinearity.

#### Characteristics of Individuals at High Risk of OSA Associated With New Mental Health Conditions (Exploratory)

To address our second objective (to describe risk profiles), we performed 2 subgroup analyses: one for individuals with a high risk of OSA without concurrent mental health conditions at baseline (conventional multivariable logistic regression) and another for individuals with a high risk of OSA regardless of mental health status (mixed multivariable logistic regression). Given the exploratory nature of this objective, we reused the same final models developed for primary analyses.

## Results

The study included 30 097 individuals from the baseline cohort (median age, 62 years [IQR, 54-71 years]; 15 320 women [50.9%] and 14 777 men [49.1%]; 89 Arab [0.3%], 221 Black [0.7%], 212 Chinese [0.7%], 47 Filipino [0.2%], 37 Japanese [0.1%], 42 Korean or West Asian [0.1%], 103 Latin American [0.3%], 271 South Asian [0.9%], 52 Southeast Asian [0.2%], 28 372 White [94.3%], 174 other racial or ethnic origin [0.6%], and 447 multiple racial or ethnic origins [1.5%]) and 27 765 individuals from the follow-up cohort (median age, 65 years [IQR, 57-73 years]; 14 133 women [50.9%] and 13 632 men [49.1%]; data on race and ethnicity collected only at baseline), with a median follow-up of 2.9 years (IQR, 2.8-3.1 years) (eTable 2 and eTable 3 in Supplement 1). Most of the participants were from urban areas (25 066 [83.3%]), were high school graduates (26 847 [89.2%]), had at least 1 chronic condition (28 121 [93.4%] at baseline and 25 711 [92.6%] at follow-up), and reported having consumed alcohol in their lifetime (29 383 [97.6%] at baseline and 27 033 [97.4%] at follow-up) (Table 1; eTable 3 in Supplement 1).

A total of 7066 of 30 097 individuals (23.5%) at baseline and 7493 of 27 765 individuals (27.0%) at follow-up were at high risk of OSA, with 4245 of 30 097 (14.1%) at baseline and 4673 of 27 765 (16.8%) at follow-up reporting witnessed apnea. The composite outcome of poor mental health was identified in 10 334 of 30 097 individuals (34.3%) at baseline, with mood disorder (5144 [17.1%]) and clinical depression (4919 [16.3%]) being the most prevalent (eTable 4 in Supplement 1); the mean (SD) CES-D-10 score was 5.3 (4.7), and the mean (SD) K10 score was 14.3 (4.6). Similarly, at follow-up, 8851 of 27 765 individuals (31.9%) met criteria for the composite outcome (eTable 4 in Supplement 1). Among individuals who did not meet criteria for the composite outcome at baseline (n = 19 990), 1372 (6.9%) met criteria for poor mental health at follow-up.

### Primary Analyses

**Figure 1.**
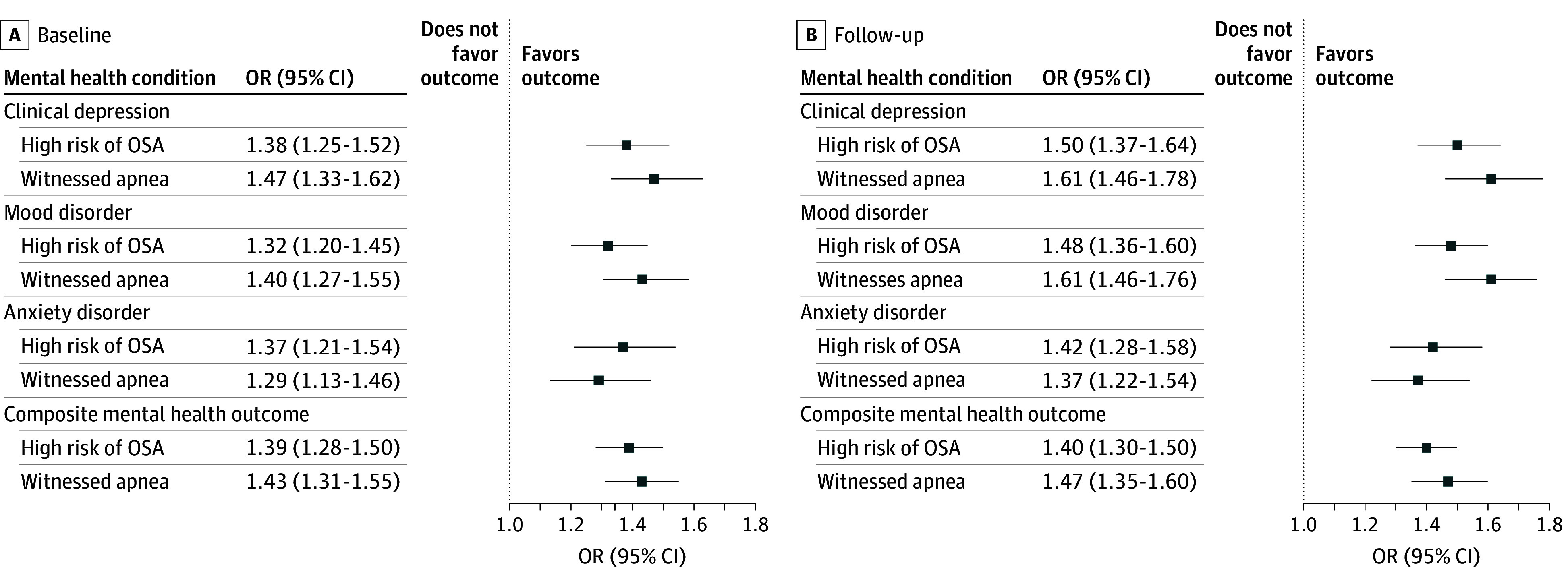
Cross-Sectional Associations at Baseline and Follow-Up of High Risk of Obstructive Sleep Apnea (OSA) and Witnessed Apnea During Sleep and Mental Health Outcomes There were 30 097 participants at baseline and 27 765 participants at follow-up. OR indicates odds ratio.

After adjustment for confounders in cross-sectional analyses, being at high risk of OSA was significantly associated with an approximately 40% increase in the odds of the composite mental health outcome (primary) at both baseline (odds ratio [OR], 1.39; 95% CI, 1.28-1.50) and follow-up (OR, 1.40; 95% CI, 1.30-1.50) (Figure 1; Table 2).

**Table 2.** Multivariable Association Between Exposures and the Composite Poor Mental Health Outcome

Exposure	Cross-sectional associations					Longitudinal associations, free from the composite outcome at baseline			Repeated-measures analysis, mixed regression^a^	
	Baseline		Follow-up							
	OR (95% CI)	Total No.	OR (95% CI)	Total No.	OR (95% CI)		Total No.	OR (95% CI)		Total No.
**Composite poor mental health outcome**										
High risk of OSA	1.39 (1.28-1.50)	23 465	1.40 (1.30-1.50)	21 240	1.20 (1.03-1.40)		14 485	1.44 (1.34-1.53)		32 420
Witnessed apnea during sleep	1.43 (1.31-1.55)	24 156	1.47 (1.35-1.60)	22 217	1.32 (1.13-1.56)		14 878	1.47 (1.37-1.57)		34 730
**Separate components**										
Anxiety disorder										
High risk of OSA	1.37 (1.21-1.54)	23 612	1.42 (1.28-1.58)	21 344	1.37 (0.92-2.05)		14 570	1.48 (1.34-1.64)		32 778
Witnessed apnea during sleep	1.29 (1.13-1.46)	24 313	1.37 (1.22-1.54)	22 324	1.32 (0.87-2.02)		14 964	1.36 (1.23-1.51)		35 120
Mood disorder										
High risk of OSA	1.32 (1.20-1.45)	23 624	1.48 (1.36-1.60)	21 344	1.65 (1.24-2.20)		14 569	1.46 (1.35-1.58)		32 804
Witnessed apnea during sleep	1.40 (1.27-1.55)	24 321	1.61 (1.46-1.76)	22 323	1.75 (1.32-2.34)		14 963	1.50 (1.39-1.62)		35 134
Clinical depression										
High risk of OSA	1.38 (1.25-1.52)	23 598	1.50 (1.37-1.64)	21 272	1.71 (1.17-2.49)		14 562	1.48 (1.36-1.61)		32 638
Witnessed apnea during sleep	1.47 (1.33-1.63)	24 298	1.61 (1.46-1.78)	22 246	1.76 (1.20-2.58)		14 954	1.51 (1.39-1.64)		34 968

Abbreviations: OR, odds ratio; OSA, obstructive sleep apnea.

^a^ The number for mixed regression presents the number of time points used in the analysis.

**Figure 2.**
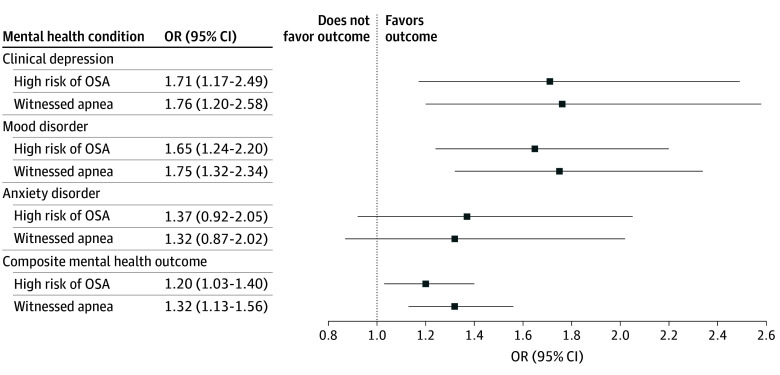
Longitudinal Associations of High Risk of Obstructive Sleep Apnea (OSA) and Witnessed Apnea During Sleep and Mental Health Outcomes Among Individuals Without Self-Reported Mental Health Conditions at Baseline There were 19 990 participants. OR indicates odds ratio.

**Figure 3.**
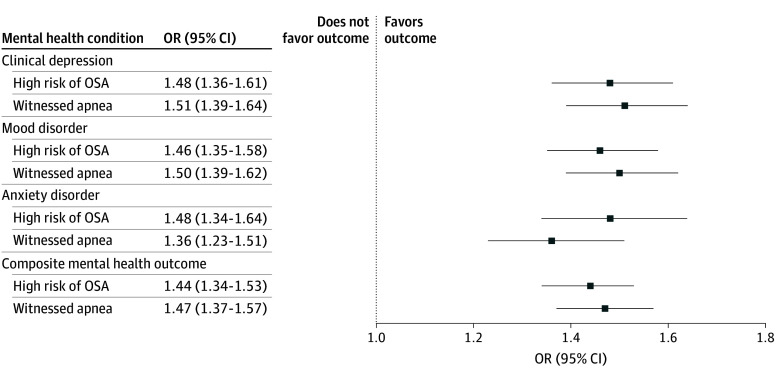
Repeated-Measures Analysis on the Associations of High Risk of Obstructive Sleep Apnea (OSA) and Witnessed Apnea During Sleep and Mental Health Outcomes Analysis was conducted on the merged baseline and follow-up cohorts; available data on 2 time points were used in multivariable mixed regressions. OR indicates odds ratio.

Among those not meeting criteria for poor mental health at baseline, after adjustment for confounders, high risk of OSA was associated with a 20% increase in the odds (OR, 1.20; 95% CI, 1.03-1.40) of developing the mental health outcome at follow-up (Figure 2; Table 2). In a repeated-measures analysis adjusted for confounders, high risk of OSA remained significantly associated with a 44% increase in the odds (OR, 1.44; 95% CI, 1.34-1.53) of the composite mental health outcome (Figure 3; Table 2).

### Secondary Analyses

We confirmed the findings using the secondary exposure definition (witnessed apnea) and secondary outcomes (mental health conditions considered separately) (Figures 1-3; Table 2). In a repeated-measures analysis, modest but statistically significant associations were noted between primary and secondary exposures and worsening mental health symptoms over time as measured by the CES-D-10 or K10 (eTable 5 in Supplement 1).

In sensitivity analyses, including all candidate variables and interaction terms, the results remained similar after adjustment, and interaction terms were not statistically significant, although findings suggested a potentially stronger association between high risk of OSA and a composite mental health outcome among women than men (eTable 6 in Supplement 1).

### Characteristics of Individuals at High Risk of OSA Associated With New Mental Health Conditions

Among 3213 participants at high risk of OSA without any mental health condition at baseline, 360 (11.2%) had new composite mental health conditions at follow-up. In multivariable analyses (eTable 7 in Supplement 1) female sex, low total household income (<$50 000), being dissatisfied or neutral with life (vs satisfied or extremely satisfied), fair self-rated general health (vs excellent), and other sleep disorders (restless legs, acting out on dreams, and insomnia symptoms) were associated with higher odds of the new composite mental health outcome at follow-up.

In a repeated-measures analysis, for individuals at high risk of OSA regardless of mental health status, in addition to the variables listed, younger age, living in an apartment (vs house), no alcohol consumption (vs once a week only), lower BMI, respiratory problems, traumatic brain injury, experiencing pain (with a dose-response association for severity), and a higher number of medications taken were significantly associated with worsening on the composite mental health outcome from baseline to follow-up (eTable 7 in Supplement 1).

## Discussion

In this national prospective cohort study, across all mental health outcomes, individuals at high risk of OSA consistently had higher odds of reported poor mental health outcomes, both cross-sectionally and longitudinally, with similar results noted for secondary exposure (ie, witnessed apnea) and outcome definitions (ie, individual mental health indices reflective of psychological distress, anxiety disorders, and mood disorders). Expanding previous knowledge based mostly on cross-sectional data, our findings strengthen the notion of OSA risk recognition for mental health.

The associations between OSA risk and mental health were moderate in strength but consistent across outcomes and analytic approaches. Although significant associations were observed for general psychological distress (K10) and anxiety disorder, the strongest associations appear to be with self-reported mood disorders and clinical depression. This study provides novel longitudinal evidence linking high risk of OSA with evolving anxiety and mood disorders. Although more detailed studies are needed, our findings suggest OSA may influence depressive states in older adults, potentially through its association with cardiovascular health, a known risk factor for depression in this age group.^50^

To our knowledge, this is one of the largest (>30 000) and most comprehensive community-based studies^51,52,53,54^ to examine the longitudinal association between OSA risk and mental health, focusing on middle-aged and older adults with comprehensive adjustment for confounders. Previous studies in this age group (n = 350-1021)^55,56^ linked OSA with concurrent depression but did not explore its role in the development of new mental health conditions. Similar to most previous studies in the general adult population,^51,52,53,54^ we found high risk of OSA to be independently associated with subsequent depression and mood disorders in older adults, although with slightly lower odds, likely due to using high risk of OSA rather than AHI-based diagnoses and adjusting for more confounders. Previous research also suggests lower odds among older adults compared with younger groups,^51,52^ which may indicate that while OSA may be associated with poor mental health in this population, other factors also play a role.

Hypoxemia, sleep fragmentation, and inflammation are potential pathways linking untreated OSA with mental health conditions.^18^ OSA-related cumulative hypoxemia can disrupt brain systems involved in mood regulation,^57,58,59^ while sleep fragmentation may alter neuroendocrine pathways.^57^ OSA is also associated with elevated inflammatory markers,^60^ which may contribute to depression.^61^ In addition, OSA-related cardiometabolic comorbidities may elevate mental distress.^62^

Several factors may interact with OSA to further increase the risk of poor mental health. In our study population, higher odds of new mental health conditions were associated with factors largely beyond an individual’s control, including female sex, younger age (also previously reported in other studies),^51,63^ lower income, poorer health and life satisfaction, less spacious living arrangements, and history of traumatic brain injury. We also identified factors, such as respiratory problems, pain, other sleep disorders, and polypharmacy, that may offer potential intervention targets for improving mental health outcomes. We did not confirm previous findings that obesity was associated with increased depression risk in OSA^53^; instead, lower BMI appeared to be associated with greater risk, possibly reflecting the older population in our study. We also found higher odds of adverse mental health in nondrinkers with OSA, although small numbers and limited data on alcohol intake make this finding difficult to interpret. Pain, mental health, and OSA are intertwined. Depression and anxiety can amplify pain perception, while chronic pain is associated with increased risks of mental health conditions.^64^ OSA has also been associated with pain severity, with OSA therapy potentially improving pain.^65,66^ This underscores the need for integrated care approaches to address these overlapping conditions.

These findings highlight the importance of systematic mental health screening for older adults at risk for OSA. Incorporating mental health assessment tools into sleep evaluation may help identify individuals at greatest risk and support early intervention.^67^ Educating older adults about the potential associations of untreated OSA with mood, cognition, and long-term brain health could also improve engagement in diagnostic testing and treatment adherence. As OSA has been associated with increased risk of cognitive impairment and dementia,^68,69^ future studies should explore whether integrating screening and prevention strategies^70^ enhances both mental and cognitive health outcomes. Research is also needed to determine whether addressing modifiable characteristics (such as respiratory problems, pain, other sleep disorders, and polypharmacy) observed among individuals at high risk of OSA who develop new mental health conditions is associated with improved mental health in this group, and to further explore possible sex-related differences suggested by our findings. Identifying high-risk subgroups may help target mental health prevention efforts in resource-limited settings. This is critical, as individuals with mental disorders are often underserved in health care,^71,72^ and those with OSA and psychiatric conditions are more likely to report unmet mental health needs despite higher service use.^73^

### Strengths and Limitations

Leveraging the CLSA, this study had several strengths, including (1) national-based sampling, with the OSA risk distribution consistent with published population-based studies^5,74,75^; (2) prospective assessment of variables of interest; (3) rich details on sleep-related variables beyond OSA risks; (4) mental health conditions assessed using validated scales; and (5) a comprehensive set of covariates, including physical activity, life satisfaction, and social support, that is usually not available in health administrative databases used for population-based and national studies.

This study also has some limitations. As with any observational study, residual confounding is possible,^76^ despite adjusting for potential confounders, and the associations observed should not be interpreted as evidence of causality. Although longitudinal and repeated-measures designs were used, the possibility of reverse causality (eg, depression contributing to OSA risk via weight gain or medication effects) remains. OSA risk and mental health diagnoses were self-reported without physician assessment or sleep tests. However, we used additional measures of mental health and OSA risk to confirm findings. Our primary exposure, the STOP questionnaire, has high sensitivity but modest specificity compared with the AHI.^28^ As a complementary secondary exposure, the witnessed apnea question provided greater specificity but lower sensitivity. This balance may have reduced misclassification and likely biased results toward the null, leading to conservative effect estimates. The AHI itself has limitations as a criterion standard, as it does not fully capture symptom burden or adverse health outcomes.^77^ Because treatment data were not available, we cannot address whether OSA therapy modifies the risk of mental health disorders. Bias from loss to follow-up and recall is also possible. Finally, the generalizability and representativeness of our study could be affected by the CLSA study design. CLSA participants were not fully representative of the Canadian population; they were predominantly White, healthier, more educated, and from urban areas, limiting finding generalizability to community-dwelling middle-aged and older adults.^26^ Complete-case analyses may introduce selection bias due to exclusion of participants with missing data, although baseline comparisons suggest minimal association with results (eTable 8 in Supplement 1).

## Conclusions

In this national cohort study, we found that middle-aged and older individuals classified as being at high risk of OSA consistently show higher odds of poor mental health than those at low risk of OSA, both cross-sectionally and longitudinally. Findings from our study address knowledge gaps regarding the association between high risk of OSA and mental health and provide valuable information for future intervention studies to develop and evaluate screening programs to protect the mental health of older adults at high risk of OSA.

## Notes

Supplement 1. **eMethods.** Detailed Inclusion and Exclusion Criteria and Data Quality Control as Per Protocol for the Canadian Longitudinal Study on Aging (CLSA) **eTable 1.** Details on Variables Extracted From the Canadian Longitudinal Study on Aging (CLSA) Databases **eTable 2.** Flow of Participants and Derivation of Final Analytic Samples Across Study Analyses **eTable 3.** Population Characteristics of Variables Considered in the Statistical Model at Baseline and Follow-Up **eTable 4.** The Distribution of the Composite Poor Mental Health Outcome and Its Components at Baseline and Follow-Up **eTable 5.** The Association Between Exposures and Changes in the Center for Epidemiologic Studies Short Depression Scale (CESD-10) or Kessler Psychological Distress Scale (K10) Over Time, Considered Separately as Continuous Variables **eTable 6.** The Association Between the High Obstructive Sleep Apnea (OSA) Risk and the Composite Poor Mental Health Outcome in the Fully Adjusted Statistical Model With and Without the Interaction Terms **eTable 7.** Characteristics of Individuals With High Obstructive Sleep Apnea (OSA) Risk Associated With New Mental Health Conditions **eTable 8.** Comparison of the Population Characteristics at Baseline of Individuals Excluded at the Follow-Up (ie, Data Available at Baseline Only) and Individuals With Available Data at Both Time Points (Baseline and Follow-Up)
eReferences.

Supplement 2.
Data Sharing Statement

## References

[1] Fan Y, Fan A, Yang Z, Fan D. Global burden of mental disorders in 204 countries and territories, 1990-2021: results from the Global Burden of Disease Study 2021. BMC Psychiatry. 2025;25(1):486. doi:10.1186/s12888-025-06932-y 40375174 PMC12080068

[2] Mental health by the numbers. National Alliance on Mental Illness. Updated April 2023. Accessed January 3, 2025. https://www.nami.org/about-mental-illness/mental-health-by-the-numbers/

[3] Jacobs P. The cost of mental health and substance abuse services in Canada: a report to the Mental Health Commission of Canada. Institute of Health Economics; 2010. Accessed November 7, 2025. https://books.scholarsportal.info/uri/ebooks/ebooks0/gibson_cppc/2010-11-18/1/10418510

[4] Leung RS, Comondore VR, Ryan CM, Stevens D. Mechanisms of sleep-disordered breathing: causes and consequences. Pflugers Arch. 2012;463(1):213-230. doi:10.1007/s00424-011-1055-x 22083643

[5] Benjafield AV, Ayas NT, Eastwood PR, . Estimation of the global prevalence and burden of obstructive sleep apnoea: a literature-based analysis. Lancet Respir Med. 2019;7(8):687-698. doi:10.1016/S2213-2600(19)30198-5 31300334 PMC7007763

[6] Punjabi NM. The epidemiology of adult obstructive sleep apnea. Proc Am Thorac Soc. 2008;5(2):136-143. doi:10.1513/pats.200709-155MG 18250205 PMC2645248

[7] Young T, Evans L, Finn L, Palta M. Estimation of the clinically diagnosed proportion of sleep apnea syndrome in middle-aged men and women. Sleep. 1997;20(9):705-706. doi:10.1093/sleep/20.9.705 9406321

[8] Jordan AS, McSharry DG, Malhotra A. Adult obstructive sleep apnoea. Lancet. 2014;383(9918):736-747. doi:10.1016/S0140-6736(13)60734-5 23910433 PMC3909558

[9] Albarrak M, Banno K, Sabbagh AA, . Utilization of healthcare resources in obstructive sleep apnea syndrome: a 5-year follow-up study in men using CPAP. Sleep. 2005;28(10):1306-1311. doi:10.1093/sleep/28.10.1306 16295216

[10] Tarasiuk A, Greenberg-Dotan S, Brin YS, Simon T, Tal A, Reuveni H. Determinants affecting health-care utilization in obstructive sleep apnea syndrome patients. Chest. 2005;128(3):1310-1314. doi:10.1378/chest.128.3.1310 16162723

[11] Kendzerska T, Mollayeva T, Gershon AS, Leung RS, Hawker G, Tomlinson G. Untreated obstructive sleep apnea and the risk for serious long-term adverse outcomes: a systematic review. Sleep Med Rev. 2014;18(1):49-59. doi:10.1016/j.smrv.2013.01.003 23642349

[12] Kendzerska T, Gershon AS, Hawker G, Leung RS, Tomlinson G. Obstructive sleep apnea and risk of cardiovascular events and all-cause mortality: a decade-long historical cohort study. PLoS Med. 2014;11(2):e1001599. doi:10.1371/journal.pmed.1001599 24503600 PMC3913558

[13] Punjabi NM, Shahar E, Redline S, Gottlieb DJ, Givelber R, Resnick HE; Sleep Heart Health Study Investigators. Sleep-disordered breathing, glucose intolerance, and insulin resistance: the Sleep Heart Health Study. Am J Epidemiol. 2004;160(6):521-530. doi:10.1093/aje/kwh261 15353412

[14] Tietjens JR, Claman D, Kezirian EJ, . Obstructive sleep apnea in cardiovascular disease: a review of the literature and proposed multidisciplinary clinical management strategy. J Am Heart Assoc. 2019;8(1):e010440. doi:10.1161/JAHA.118.010440 30590966 PMC6405725

[15] Luzzi V, Mazur M, Guaragna M, . Correlations of obstructive sleep apnea syndrome and daytime sleepiness with the risk of car accidents in adult working population: a systematic review and meta-analysis with a gender-based approach. J Clin Med. 2022;11(14):3971. doi:10.3390/jcm11143971 35887735 PMC9319534

[16] Garbarino S, Guglielmi O, Sanna A, Mancardi GL, Magnavita N. Risk of occupational accidents in workers with obstructive sleep apnea: systematic review and meta-analysis. Sleep. 2016;39(6):1211-1218. doi:10.5665/sleep.5834 26951401 PMC4863208

[17] Pendharkar SR, Kaambwa B, Kapur VK. The cost-effectiveness of sleep apnea management: a critical evaluation of the impact of therapy on health care costs. Chest. 2024;166(3):612-621. doi:10.1016/j.chest.2024.04.024 38815624

[18] BaHammam AS, Kendzerska T, Gupta R, et al. Comorbid depression in obstructive sleep apnea: an under-recognized association. Sleep Breath. 2016;20(2):447-456. doi:10.1007/s11325-015-1223-x 26156890

[19] Haba-Rubio J. Psychiatric aspects of organic sleep disorders. Dialogues Clin Neurosci. 2005;7(4):335-346. doi:10.31887/DCNS.2005.7.4/jhabarubio 16416709 PMC3181746

[20] Gupta MA, Simpson FC. Obstructive sleep apnea and psychiatric disorders: a systematic review. J Clin Sleep Med. 2015;11(2):165-175. doi:10.5664/jcsm.4466 25406268 PMC4298774

[21] Edwards C, Almeida OP, Ford AH. Obstructive sleep apnea and depression: a systematic review and meta-analysis. Maturitas. 2020;142:45-54. doi:10.1016/j.maturitas.2020.06.002 33158487

[22] Alam A, Chengappa KN, Ghinassi F. Screening for obstructive sleep apnea among individuals with severe mental illness at a primary care clinic. Gen Hosp Psychiatry. 2012;34(6):660-664. doi:10.1016/j.genhosppsych.2012.06.015 22832135

[23] Hattori M, Kitajima T, Mekata T, . Risk factors for obstructive sleep apnea syndrome screening in mood disorder patients. Psychiatry Clin Neurosci. 2009;63(3):385-391. doi:10.1111/j.1440-1819.2009.01956.x 19566771

[24] Canadian Longitudinal Study on Aging. Accessed November 4, 2025. http://www.clsa-elcv.ca/

[25] Raina PS, Wolfson C, Kirkland SA, . The Canadian Longitudinal Study on Aging (CLSA). Can J Aging. 2009;28(3):221-229. doi:10.1017/S0714980809990055 19860977

[26] Raina P, Wolfson C, Kirkland S, . Cohort profile: the Canadian Longitudinal Study on Aging (CLSA). Int J Epidemiol. 2019;48(6):1752-1753. doi:10.1093/ije/dyz173 31633757 PMC6929533

[27] von Elm E, Altman DG, Egger M, Pocock SJ, Gøtzsche PC, Vandenbroucke JP; STROBE Initiative. The Strengthening the Reporting of Observational Studies in Epidemiology (STROBE) statement: guidelines for reporting observational studies. J Clin Epidemiol. 2008;61(4):344-349. doi:10.1016/j.jclinepi.2007.11.008 18313558

[28] Chung F, Yegneswaran B, Liao P, . STOP questionnaire: a tool to screen patients for obstructive sleep apnea. Anesthesiology. 2008;108(5):812-821. doi:10.1097/ALN.0b013e31816d83e4 18431116

[29] Patel D, Tsang J, Saripella A, . Validation of the STOP questionnaire as a screening tool for OSA among different populations: a systematic review and meta-regression analysis. J Clin Sleep Med. 2022;18(5):1441-1453. doi:10.5664/jcsm.9820 34910625 PMC9059595

[30] Chiu HY, Chen PY, Chuang LP, . Diagnostic accuracy of the Berlin questionnaire, STOP-BANG, STOP, and Epworth Sleepiness Scale in detecting obstructive sleep apnea: a bivariate meta-analysis. Sleep Med Rev. 2017;36:57-70. doi:10.1016/j.smrv.2016.10.004 27919588

[31] Li X, Zha L, Zhou L, . Diagnostic utility of obstructive sleep apnea screening questionnaires: a comprehensive meta-analysis. Sleep Breath. 2024;29(1):14. doi:10.1007/s11325-024-03169-z 39601864

[32] Kapur VK, Auckley DH, Chowdhuri S, . Clinical practice guideline for diagnostic testing for adult obstructive sleep apnea: an American Academy of Sleep Medicine clinical practice guideline. J Clin Sleep Med. 2017;13(3):479-504. doi:10.5664/jcsm.6506 28162150 PMC5337595

[33] Bernhardt L, Brady EM, Freeman SC, . Diagnostic accuracy of screening questionnaires for obstructive sleep apnoea in adults in different clinical cohorts: a systematic review and meta-analysis. Sleep Breath. 2022;26(3):1053-1078. doi:10.1007/s11325-021-02450-9 34406554 PMC8370860

[34] Feltner C, Wallace IF, Aymes S, . Screening for obstructive sleep apnea in adults: updated evidence report and systematic review for the US Preventive Services Task Force. JAMA. 2022;328(19):1951-1971. doi:10.1001/jama.2022.18357 36378203

[35] Hann D, Winter K, Jacobsen P. Measurement of depressive symptoms in cancer patients: evaluation of the Center for Epidemiological Studies Depression Scale (CES-D). J Psychosom Res. 1999;46(5):437-443. doi:10.1016/S0022-3999(99)00004-5 10404478

[36] Andresen EM, Byers K, Friary J, Kosloski K, Montgomery R. Performance of the 10-item Center for Epidemiologic Studies Depression scale for caregiving research. SAGE Open Med. 2013;1:2050312113514576. doi:10.1177/2050312113514576 26770693 PMC4687763

[37] Kessler RC, Barker PR, Colpe LJ, . Screening for serious mental illness in the general population. Arch Gen Psychiatry. 2003;60(2):184-189. doi:10.1001/archpsyc.60.2.184 12578436

[38] Andresen EM, Malmgren JA, Carter WB, Patrick DL. Screening for depression in well older adults: evaluation of a short form of the CES-D (Center for Epidemiologic Studies Depression Scale). Am J Prev Med. 1994;10(2):77-84. doi:10.1016/S0749-3797(18)30622-6 8037935

[39] Irwin M, Artin KH, Oxman MN. Screening for depression in the older adult: criterion validity of the 10-item Center for Epidemiological Studies Depression Scale (CES-D). Arch Intern Med. 1999;159(15):1701-1704. doi:10.1001/archinte.159.15.1701 10448771

[40] Sampasa-Kanyinga H, Zamorski MA, Colman I. The psychometric properties of the 10-item Kessler Psychological Distress Scale (K10) in Canadian military personnel. PLoS One. 2018;13(4):e0196562. doi:10.1371/journal.pone.0196562 29698459 PMC5919406

[41] Yayan J, Rasche K. A systematic review of risk factors for sleep apnea. Prev Med Rep. 2024;42:102750. doi:10.1016/j.pmedr.2024.102750 38741931 PMC11089396

[42] Zimmermann M, Chong AK, Vechiu C, Papa A. Modifiable risk and protective factors for anxiety disorders among adults: a systematic review. Psychiatry Res. 2020;285:112705. doi:10.1016/j.psychres.2019.112705 31839417

[43] Zolfaghari S, Yao C, Thompson C, . Effects of menopause on sleep quality and sleep disorders: Canadian Longitudinal Study on Aging. Menopause. 2020;27(3):295-304. doi:10.1097/GME.0000000000001462 31851117

[44] Diener E, Emmons RA, Larsen RJ, Griffin S. The Satisfaction With Life Scale. J Pers Assess. 1985;49(1):71-75. doi:10.1207/s15327752jpa4901_13 16367493

[45] Zhao JL, Cross N, Yao CW, . Insomnia disorder increases the risk of subjective memory decline in middle-aged and older adults: a longitudinal analysis of the Canadian Longitudinal Study on Aging. Sleep. 2022;45(11):zsac176. doi:10.1093/sleep/zsac176 35877203 PMC9644124

[46] Bastien CH, Vallières A, Morin CM. Validation of the Insomnia Severity Index as an outcome measure for insomnia research. Sleep Med. 2001;2(4):297-307. doi:10.1016/S1389-9457(00)00065-4 11438246

[47] Buysse DJ, Reynolds CF III, Monk TH, Berman SR, Kupfer DJ. The Pittsburgh Sleep Quality Index: a new instrument for psychiatric practice and research. Psychiatry Res. 1989;28(2):193-213. doi:10.1016/0165-1781(89)90047-4 2748771

[48] Raina PS, Wolfson C, Kirkland SA, . Ascertainment of chronic diseases in the Canadian Longitudinal Study on Aging (CLSA), systematic review. Can J Aging. 2009;28(3):275-285. doi:10.1017/S071498080999002X 19860982

[49] Ambler G, Brady AR, Royston P. Simplifying a prognostic model: a simulation study based on clinical data. Stat Med. 2002;21(24):3803-3822. doi:10.1002/sim.1422 12483768

[50] Zhang Y, Chen Y, Ma L. Depression and cardiovascular disease in elderly: current understanding. J Clin Neurosci. 2018;47:1-5. doi:10.1016/j.jocn.2017.09.022 29066229

[51] Chen YH, Keller JK, Kang JH, Hsieh HJ, Lin HC. Obstructive sleep apnea and the subsequent risk of depressive disorder: a population-based follow-up study. J Clin Sleep Med. 2013;9(5):417-423. doi:10.5664/jcsm.2652 23674930 PMC3629313

[52] Lu MK, Tan HP, Tsai IN, Huang LC, Liao XM, Lin SH. Sleep apnea is associated with an increased risk of mood disorders: a population-based cohort study. Sleep Breath. 2017;21(2):243-253. doi:10.1007/s11325-016-1389-x 27495797

[53] LaGrotte C, Fernandez-Mendoza J, Calhoun SL, Liao D, Bixler EO, Vgontzas AN. The relative association of obstructive sleep apnea, obesity and excessive daytime sleepiness with incident depression: a longitudinal, population-based study. Int J Obes (Lond). 2016;40(9):1397-1404. doi:10.1038/ijo.2016.87 27143032 PMC5014694

[54] Pan ML, Tsao HM, Hsu CC, . Bidirectional association between obstructive sleep apnea and depression: a population-based longitudinal study. Medicine (Baltimore). 2016;95(37):e4833. doi:10.1097/MD.0000000000004833 27631236 PMC5402579

[55] Xue X, Qian K, Zhao LB, . The connection between depression and frailty among older adults with obstructive sleep apnea: results from a multicenter cohort study. Sleep Breath. 2025;29(1):114. doi:10.1007/s11325-025-03271-w 40014277

[56] Farajzadeh M, Hosseini M, Mohtashami J, Chaibakhsh S, Zagheri Tafreshi M, Ghanei Gheshlagh R. The association between obstructive sleep apnea and depression in older adults. Nurs Midwifery Stud. 2016;5(2):e32585. doi:10.17795/nmsjournal32585 27579333 PMC5002089

[57] Meerlo P, Sgoifo A, Suchecki D. Restricted and disrupted sleep: effects on autonomic function, neuroendocrine stress systems and stress responsivity. Sleep Med Rev. 2008;12(3):197-210. doi:10.1016/j.smrv.2007.07.007 18222099

[58] Rosenzweig I, Glasser M, Crum WR, . Changes in neurocognitive architecture in patients with obstructive sleep apnea treated with continuous positive airway pressure. EBioMedicine. 2016;7:221-229. doi:10.1016/j.ebiom.2016.03.020 27322475 PMC4909326

[59] Song X, Roy B, Kang DW, . Altered resting-state hippocampal and caudate functional networks in patients with obstructive sleep apnea. Brain Behav. 2018;8(6):e00994. doi:10.1002/brb3.994 29749715 PMC5991585

[60] Hirsch Allen AJ, Kendzerska T, Bhatti P, . Obstructive sleep apnea severity, circulating biomarkers, and cancer risk. J Clin Sleep Med. 2024;20(9):1415-1422. doi:10.5664/jcsm.11170 38648119 PMC11367715

[61] Dowlati Y, Herrmann N, Swardfager W, . A meta-analysis of cytokines in major depression. Biol Psychiatry. 2010;67(5):446-457. doi:10.1016/j.biopsych.2009.09.033 20015486

[62] Garbarino S, Bardwell WA, Guglielmi O, Chiorri C, Bonanni E, Magnavita N. Association of anxiety and depression in obstructive sleep apnea patients: a systematic review and meta-analysis. Behav Sleep Med. 2020;18(1):35-57. doi:10.1080/15402002.2018.1545649 30453780

[63] Mokhlesi B, Ham SA, Gozal D. The effect of sex and age on the comorbidity burden of OSA: an observational analysis from a large nationwide US health claims database. Eur Respir J. 2016;47(4):1162-1169. doi:10.1183/13993003.01618-2015 26797029

[64] Goesling J, Clauw DJ, Hassett AL. Pain and depression: an integrative review of neurobiological and psychological factors. Curr Psychiatry Rep. 2013;15(12):421. doi:10.1007/s11920-013-0421-0 24214740

[65] Charokopos A, Card ME, Gunderson C, Steffens C, Bastian LA. The association of obstructive sleep apnea and pain outcomes in adults: a systematic review. Pain Med. 2018;19(suppl 1):S69-S75. doi:10.1093/pm/pny140 30203008

[66] Shen C, Ou Y, Ouyang R, Zong D. Prevalence and characteristics of pain in moderate-to-severe obstructive sleep apnea patients and effect of CPAP treatment. Sci Rep. 2023;13(1):15758. doi:10.1038/s41598-023-42967-5 37735494 PMC10514028

[67] Velescu DR, Marc MS, Traila D, . A narrative review of self-reported scales to evaluate depression and anxiety symptoms in adult obstructive sleep apnea patients. Medicina (Kaunas). 2024;60(2):261. doi:10.3390/medicina60020261 38399548 PMC10889932

[68] Gosselin N, Baril AA, Osorio RS, Kaminska M, Carrier J. Obstructive sleep apnea and the risk of cognitive decline in older adults. Am J Respir Crit Care Med. 2019;199(2):142-148. doi:10.1164/rccm.201801-0204PP 30113864 PMC6943882

[69] Legault J, Thompson C, Moullec G, . Age- and sex-specific associations between obstructive sleep apnea risk and cognitive decline in middle-aged and older adults: a 3-year longitudinal analysis of the Canadian Longitudinal Study on Aging. Sleep Med. 2023;112:77-87. doi:10.1016/j.sleep.2023.09.029 37832163

[70] Singh V, Kumar A, Gupta S. Mental health prevention and promotion—a narrative review. Front Psychiatry. 2022;13:898009. doi:10.3389/fpsyt.2022.898009 35958637 PMC9360426

[71] Levinson Miller C, Druss BG, Dombrowski EA, Rosenheck RA. Barriers to primary medical care among patients at a community mental health center. Psychiatr Serv. 2003;54(8):1158-1160. doi:10.1176/appi.ps.54.8.1158 12883146

[72] Druss BG, Rosenheck RA. Mental disorders and access to medical care in the United States. Am J Psychiatry. 1998;155(12):1775-1777. doi:10.1176/ajp.155.12.1775 9842793

[73] Kaufmann CN, Susukida R, Depp CA. Sleep apnea, psychopathology, and mental health care. Sleep Health. 2017;3(4):244-249. doi:10.1016/j.sleh.2017.04.003 28709510 PMC5560422

[74] What is the impact of sleep apnea on Canadians? fast facts from the *2009 Canadian Community Health Survey—Sleep Apnea Rapid Response*. Goverment of Canada. Updated March 15, 2021. Accessed October 6, 2025. https://www.canada.ca/en/public-health/services/chronic-diseases/sleep-apnea/what-impact-sleep-apnea-on-canadians.html

[75] Sleep apnea in Canada, 2016 and 2017. Statistics Canada. Updated October 14, 2018. Accessed October 6, 2025. https://www150.statcan.gc.ca/n1/pub/82-625-x/2018001/article/54979-eng.htm

[76] Lin DY, Psaty BM, Kronmal RA. Assessing the sensitivity of regression results to unmeasured confounders in observational studies. Biometrics. 1998;54(3):948-963. doi:10.2307/2533848 9750244

[77] Cao W, Luo J, Xiao Y. A review of current tools used for evaluating the severity of obstructive sleep apnea. Nat Sci Sleep. 2020;12:1023-1031. doi:10.2147/NSS.S275252 33239929 PMC7680675

