# Supplementary material for: Obstructive Sleep Apnea Risk and Mental Health Conditions Among Older Canadian Adults in the Canadian Longitudinal Study on Aging
Source: JAMA Netw Open. 2025 Dec 26;8(12):e2549137. doi: 10.1001/jamanetworkopen.2025.49137 (PMC12743280; doi:10.1001/jamanetworkopen.2025.49137)
Supplement: Supplement 1. — eMethods. Detailed Inclusion and Exclusion Criteria and Data Quality Control as Per Protocol for the Canadian Longitudinal Study on Aging (CLSA) eTable 1. Details on Variables Extracted From the Canadian Longitudinal Study on Aging (CLSA) Databases eTable 2. Flow of Participants and Derivation of Final Analytic Samples Across Study Analyses eTable 3. Population Characteristics of Variables Considered in the Statistical Model at Baseline and Follow-Up eTable 4. The Distribution of the Composite Poor Mental Health Outcome and Its Components at Baseline and Follow-Up eTable 5. The Association Between Exposures and Changes in the Center for Epidemiologic Studies Short Depression Scale (CESD-10) or Kessler Psychological Distress Scale (K10) Over Time, Considered Separately as Continuous Variables eTable 6. The Association Between the High Obstructive Sleep Apnea (OSA) Risk and the Composite Poor Mental Health Outcome in the Fully Adjusted Statistical Model With and Without the Interaction Terms eTable 7. Characteristics of Individuals With High Obstructive Sleep Apnea (OSA) Risk Associated With New Mental Health Conditions eTable 8. Comparison of the Population Characteristics at Baseline of Individuals Excluded at the Follow-Up (ie, Data Available at Baseline Only) and Individuals With Available Data at Both Time Points (Baseline and Follow-Up) eReferences. [file jamanetwopen-e2549137-s001.pdf]

Supplementary Online Content

Kendzerska T, Mallick R, Li W, et al. Obstructive sleep apnea risk and mental health conditions among older Canadian adults in the Canadian Longitudinal Study on Aging. *JAMA Netw Open.* 2025;8(12): 2549137. doi:10.1001/jamanetworkopen.2025.49137

eMethods. Detailed Inclusion and Exclusion Criteria and Data Quality Control as Per Protocol for the Canadian Longitudinal Study on Aging (CLSA)
eTable 1. Details on Variables Extracted From the Canadian Longitudinal Study on Aging (CLSA) Databases
eTable 2. Flow of Participants and Derivation of Final Analytic Samples Across Study Analyses
eTable 3. Population Characteristics of Variables Considered in the Statistical Model at Baseline and Follow-Up
eTable 4. The Distribution of the Composite Poor Mental Health Outcome and Its Components at Baseline and Follow-Up
eTable 5. The Association Between Exposures and Changes in the Center for Epidemiologic Studies Short Depression Scale (CESD-10) or Kessler Psychological Distress Scale (K10) Over Time, Considered Separately as Continuous Variables
eTable 6. The Association Between the High Obstructive Sleep Apnea (OSA) Risk and the Composite Poor Mental Health Outcome in the Fully Adjusted Statistical Model With and Without the Interaction Terms
eTable 7. Characteristics of Individuals With High Obstructive Sleep Apnea (OSA) Risk Associated With New Mental Health Conditions
eTable 8. Comparison of the Population Characteristics at Baseline of Individuals Excluded at the Follow-Up (ie, Data Available at Baseline Only) and Individuals With Available Data at Both Time Points (Baseline and Follow-Up)
eReferences.

This supplementary material has been provided by the authors to give readers additional information about their work.

eMethods. Detailed Inclusion and Exclusion Criteria and Data Quality Control as per Protocol for the Canadian Longitudinal Study on Aging (CLSA)

Inclusion and exclusion criteria: Details on inclusion and exclusion criteria for the CLSA are available at: www.clsa-elcv.ca/wp-content/uploads/2023/06/combinedprotocol_v3_2013_for_web.pdf. To summarize, excluded from the sampling frame, and consequently, the CLSA are residents in the three territories and some remote regions, persons living on federal First Nations reserves and other First Nations settlements in the provinces, full-time members of the Canadian Armed Forces, and individuals living in institutions. This latter exclusion means that individuals living in long-term care institutions (i.e., those providing 24-hour nursing care) are excluded from the CLSA at baseline. Individuals living in households and transitional housing arrangements (e.g., seniors' residences, in which only minimal care is provided) are included at baseline. Additionally, when sampling from Ministry of Health registration databases, individuals who were temporary visa holders or had transitional health coverage (when available) were excluded, as this represented a more transient population that may be difficult to follow in a long-term study. Participants who become institutionalized during the course of follow-up remain in the CLSA and will continue to be followed through either personal interview or interviews with proxies.

Individuals unable to respond in English or French are excluded from the CLSA. The presence of chronic medical illness at baseline is not a reason for exclusion from the study. However, individuals with cognitive impairment at baseline are excluded from the study. The presence of cognitive impairment not only potentially compromises the capacity to give informed consent, but may also affect the reliability and validity of interview responses.

***Data quality control*:** Details on data quality control for the CLSA are available at: www.clsa-elcv.ca/wp-content/uploads/2023/06/combinedprotocol_v3_2013_for_web.pdf. To summarize, each site is responsible for verifying the completeness and accuracy of the data collected during the interview. With participant permission, modules of the interview are recorded for quality control. Computer-Assisted Telephone Interview (CATI) managers also listen to 10% of all live interviews. The CATI software is programmed to flag missing or inconsistent information such that data queries can be made in real-time. The CLSA National Coordinating Centre (NCC) forwards the de-identified data to the Statistical Analysis Centre (SAC). The SAC checks the data for missingness, outliers, improper skip patterns, implausible values, and other anomalies. The SAC links subsequent waves of data based on unique identifiers and does not have access to identifying information, such as names or contact information. Having all data centrally located at the NCC during data collection ensures maximal data security, efficient management, and quality control.

Details on a mixed regression model

Among individuals with information available at both baseline and follow-up, we examined the association between high OSA risk and mental health outcomes using mixed-effects multivariable logistic regression models. These models incorporated data from both time points, allowing each participant to contribute multiple observations over time. To account for within-subject correlation arising from repeated measurements of the same individuals, we specified a compound symmetry covariance structure, which assumes equal variances and a constant covariance (or correlation) between any pair of measurements within the same participant. Random intercepts were included to model individual-specific effects not captured by the fixed covariates.

© 2025 Kendzerska T et al. *JAMA Network Open.*

Variable selection process for the baseline*.

First, we removed variables with more than 10% missing values to preserve the sample size. Then, we excluded collinear variables based on the variable clustering algorithm. Finally, we used the step-down procedure described by Ambler et al.,[1] where variables are sequentially deleted to obtain the lowest, stable Akaike information criterion (AIC) value, selecting the final models, which included 19 variables for the baseline.

#	All variables considered, including exposure#	N Miss (%)	Clusters
	**Exposures**		
	Primary: High-risk for obstructive sleep apnea (OSA)	2,826 (9.4)	Cluster 2
	Secondary: Witnessed apnea during sleep	1,898 (6.3)	Cluster 2
	**Covariates**		
1.	Start Date Year	0 (0)	Cluster 16
2.	Age	0 (0)	Cluster 21
3.	Sex	0 (0)	Cluster 6
4.	**Married?**	**8 (0)**	**Cluster 1**
5.	Dwelling Type	10 (0)	Cluster 11
6.	**Total household income**	**1,941 (6.4)**	**Cluster 1**
7.	Education high school graduated	2,054 (6.8)	Cluster 7
8.	Self-rated general health	23 (0.1)	Cluster 24
9.	**Satisfaction with Life Scale**	**354 (1.2)**	**Cluster 17**
10.	**Cigarettes smoked current frequency**	**9,603 (31.9)**	**—**
11.	Frequency of alcohol consumption, past 12 months	726 (2.4)	Cluster 13
12.	**On average, how many hours per day did you spend walking?**	**5,731 (19)**	**—**
13.	Body Mass Index (BMI)	136 (0.5)	Cluster 14
14.	**Functional Social Support**	**606 (2)**	**Cluster 17**
15.	Cardiovascular Diseases (CVD) / Stroke	209 (0.7)	Cluster 4
16.	Hypertension	268 (0.9)	Cluster 22
17.	Diabetes	110 (0.4)	Cluster 19
18.	Respiratory Problem?	200 (0.7)	Cluster 15
19.	Ever cancer told by doctor	93 (0.3)	Cluster 9
20.	Intensity of pain or discomfort	1,505 (5)	Cluster 5
21.	Positive Screen for Traumatic Brain Injury	0 (0)	Cluster 12
22.	Underactive thyroid gland	394 (1.3)	Cluster 8
23.	# medications taken by a participant	0 (0)	Cluster 23
24.	Acts out on dreams while asleep	277 (0.9)	Cluster 10
25.	Restless Leg Syndrome	72 (0.2)	Cluster 18
26.	**Overall dissatisfaction with sleep pattern**	**24 (0.1)**	**Cluster 3**
27.	**Number of sleep hours during the past month**	**69 (0.2)**	**Cluster 3**
28.	Probable insomnia (Difficulty falling asleep OR maintaining sleep) AND functional impairment	133 (0.4)	Cluster 20

* Ethnicity and urbanicity were not considered as covariates because more than 90% of CLSA participants self-identified as White and lived in urban areas, resulting in limited variability in these measures. When a covariate shows such low prevalence (<10%) or limited distribution across exposure and outcome categories, inclusion in multivariable models may introduce instability and overfitting without meaningfully improving model adjustment.[2]

#Exposure (the OSA status) was forced into the model; primary and secondary exposure were considered separately.

In gray: variables not considered in the multivariable model as based on steps 1 and 2.

Step 1: removed variables with more than 10% missing values: "Cigarettes smoked current frequency" and "On average, how many hours per day did you spend walking?"

© 2025 Kendzerska T et al. *JAMA Network Open.*

Step 2: excluded collinear variables based on variable clustering algorithm:

- **Cluster 1:** "Married?" and "Total household income"; we kept "Total household income" as a more frequently available/used variable.
- **Cluster 3: "**Overall dissatisfaction with sleep pattern" and "Number of sleep hours during the past month"; we kept "Number of sleep hours during the past month" as a more frequently available/used variable.
- **Cluster 17:** "Satisfaction with Life Scale" and "Functional Social Support"; we kept "Satisfaction with Life Scale" as a validated scale with fewer missing values.

Step 3: The final model selection as based on the AIC value.

**Models**	**Variables excluded**	**AIC**
1 (exposure + 23 variables selected based on Steps 1 and 2)	None	23794.916
2	Education high school graduated	23793.130
3	Cardiovascular Diseases (CVD) / Stroke	23791.408
4	Start Date Year	23789.977
5	Ever cancer told by doctor	23789.953
6	Diabetes	23790.003
7	Underactive thyroid gland	23790.218
8	Body Mass Index (BMI)	23792.580
9	Frequency alcohol consumption	23795.563
10	Number of sleep hours during the past month	23799.478
11	Dwelling Type	23809.702
12	Hypertension	23820.683

The final list of variables at baseline (N=19; in grey): age, sex, dwelling type, total household income, self-rated general health, satisfaction with life, alcohol consumption, BMI, self-reported hypertension, diabetes, respiratory problem, usual intensity of pain or discomfort, positive screen for traumatic brain injury, under-active thyroid gland, the number of medications taken, self-reported acting out on dream, restless legs, number of sleep hours per night, insomnia with daytime impairment.

© 2025 Kendzerska T et al. *JAMA Network Open.*

Variable selection process for the follow-up.

First, we removed variables with more than 10% missing values to preserve the sample size. Then, we excluded collinear variables based on the variable clustering algorithm. Finally, we used the step-down procedure described by Ambler et al.,[1] where variables are sequentially deleted to obtain the lowest, stable AIC value, selecting the final models, which included 17 variables for the follow-up.

#	All variables considered, including exposure*	N Miss (%)	Clusters
	**Exposures**		
	Primary: High-risk for obstructive sleep apnea (OSA)	2,744 (9.9)	Cluster 6
	Secondary: Witnessed apnea during sleep	1,353 (4.9)	Cluster 6
	**Covariates**		
29.	Start Date Year	0 (0)	Cluster 10
30.	**Age**	**0 (0)**	**Cluster 4**
31.	Sex	0 (0)	Cluster 18
32.	**Married?**	**11 (0)**	**Cluster 1**
33.	Dwelling Type	1 (0)	Cluster 9
34.	**Total household income**	**1,771 (6.4)**	**Cluster 1**
35.	Education high school graduated	1,734 (6.2)	Cluster 7
36.	**Self-rated general health**	**38 (0.1)**	**Cluster 5**
37.	**Satisfaction with Life Scale**	356 (1.3)	Cluster 18
38.	Cigarettes smoked current frequency	29 (0.1)	Cluster 14
39.	Frequency of alcohol consumption, past 12 months	751 (2.7)	Cluster 15
40.	**On average, how many hours per day did you spend walking?**	**4,927 (17.7)**	**—**
41.	Body Mass Index (BMI)	1020 (3.7)	Cluster 2
42.	**Functional Social Support**	1356 (4.9)	Cluster 18
43.	Cardiovascular Diseases (CVD) / Stroke	1,149 (4.1)	Cluster 22
44.	Hypertension	1,509 (5.4)	Cluster 23
45.	Diabetes	1,050 (3.8)	Cluster 21
46.	Respiratory Problem?	1,146 (4.1)	Cluster 16
47.	Ever cancer told by doctor	1,036 (3.7)	Cluster 13
48.	**Intensity of pain or discomfort**	**712 (2.6)**	**Cluster 5**
49.	Positive Screen for Traumatic Brain Injury	1,143 (4.1)	Cluster 12
50.	Underactive thyroid gland	1,230 (4.4)	Cluster 8
51.	**# medications taken by a participant**	**3 (0)**	**Cluster 4**
52.	Acts out on dreams while asleep	1,333 (4.8)	Cluster 11
53.	Restless Leg Syndrome	955 (3.4)	Cluster 19
54.	**Overall dissatisfaction with sleep pattern**	**847 (3.1)**	**Cluster 3**
55.	**Number of sleep hours during the past month**	**909 (3.3)**	**Cluster 3**
56.	Probable insomnia (Difficulty falling asleep OR maintaining sleep) AND functional impairment	1,056 (3.8)	Cluster 20

*Exposure (the OSA status) was forced into the model; primary and secondary exposure were considered separately.

In gray: variables not considered in the multivariable model as based on steps 1 and 2.

Step 1: removed variables with more than 10% missing values: "On average, how many hours per day did you spend walking?"

Step 2: excluded collinear variables based on variable clustering algorithm:

- **Cluster 1:** "Married?" and "Total household income"; we kept "Total household income" as a more frequently available/used variable.
- **Cluster 3: "**Overall dissatisfaction with sleep pattern" and "Number of sleep hours during the past month"; we kept "Number of sleep hours during the past month" as a more frequently available/used variable.

© 2025 Kendzerska T et al. *JAMA Network Open.*

- **Cluster 4:** "Age" and "# medications taken by a participant"; we kept "Age" as a more frequently available/used variable.
- **Cluster 5:** "Self-rated general health" and "Intensity of pain or discomfort"; we kept " Intensity of pain or discomfort " as a more frequently available/used variable.
- **Cluster 18:** "Satisfaction with Life Scale" and "Functional Social Support"; we kept "Satisfaction with Life Scale" as a validated scale with fewer missing values.

Step 3: The final model selection as based on the AIC value.

**Models**	**Variables excluded**	**AIC**
1 (exposure + 22 variables selected based on Steps 1 and 2)	None	20927.16
2	Hypertension	20925.16
3	Body Mass Index (BMI)	20923.40
4	Start Date Year	20921.82
5	Ever cancer told by doctor	20920.38
6	Education high school graduated	20918.97
7	Current frequency cigarettes smoked	20924.32
8	Frequency alcohol consumption past 12 months	20932.69
9	Cardiovascular Diseases (CVD) / Stroke	20941.45

The final list of variables at follow-up (N=17; in grey): age, sex, dwelling type, total household income, satisfaction with life, current smoker status, alcohol consumption, self-reported cardiovascular and cerebrovascular conditions, diabetes, respiratory problem, usual intensity of pain or discomfort, positive screen for traumatic brain injury, under-active thyroid gland, self-reported acting out on dream, restless legs, number of sleep hours per night, insomnia with daytime impairment.

© 2025 Kendzerska T et al. *JAMA Network Open.*

eTable 1. Details on Variables Extracted From the Canadian Longitudinal Study on Aging (CLSA) Databases

Variables	Details
**Self-reported obstructive sleep apnea** (OSA) (collected at baseline and follow-up)	Relevant questions:<br>– Do you snore loudly? By 'loudly' I mean louder than talking or loud enough to be heard through closed doors.<br>– Has anyone ever observed you stop breathing in your sleep?<br>***Primary definition:*** Self-reported was identified by the standard validated STOP questionnaire:[3] Having at least 2 self-reported symptoms of snoring, daytime somnolence, being observed to stop breathing, or hypertension indicates a high risk of OSA. We utilized a similar approach to Zolfaghari et al.[4] Using apnea-hypopnea index (AHI) greater than 5 (i.e., indicating the presence of OSA) as a cutoff value to evaluate the STOP questionnaire, the sensitivity was 66%, the specificity was 60%, the positive predictive value (PPV) was 78%, and the negative predictive value (NPV) was 44%. A systematic review confirmed its validity (a STOP score ≥ 2) across populations, with strong discriminatory power (NPV >84%) for ruling out severe OSA (AHI ≥30) in individuals with comorbidities.[5]
**Sleep and insomnia measures**[6] (collected at baseline and follow-up)	– Measures of sleep habits were collected at baseline and follow-up through structured self-report questions that were designed for this study as part of a larger set of measures to assess physical functioning. The questions were drawn from validated sleep questionnaires[7,8] and captured important aspects of sleep and their relation to health. The questions covered six domains over the last month: (1) participants' satisfaction with the type of sleep they were getting; (2) hours of nighttime sleep; (3) trouble falling asleep or staying asleep; (4) daytime sleepiness; (5) dream enactment behavior defined as Have you ever been told, or suspected yourself, that you seem to "act out your dreams" while asleep (for example, punching, flailing your arms in the air, making running movements, etc.)?; (6) if they experienced, recurrent, uncomfortable feelings or sensations in the legs, or urges to move their legs while sitting or lying down.<br>– A probable diagnosis of insomnia disorder:[9] only participants who experienced difficulties with sleep onset or maintenance three times or more per week, for longer than 3 months, and stated that it significantly interfered (≥ "Much") with their daily functioning, and additionally were dissatisfied with their sleep pattern (< "Neutral") will be categorized as having probable insomnia disorder. Any participant who experienced difficulties with sleep onset or maintenance three times or more per week but did not report any interference (< "Much") with daytime functioning will be categorized as having insomnia symptoms only. All other participants were classified as having no insomnia symptoms.
**Mental Health Measures** (collected at baseline and follow-up)	Relevant questions:<br>– Has a doctor ever told you that you have an anxiety disorder such as a phobia, obsessive-compulsive disorder or a panic disorder?<br>– Has a doctor ever told you that you have a mood disorder such as depression (including manic depression), bipolar disorder, mania, or dysthymia?<br>– Has a doctor ever told you that you suffer from clinical depression?<br>– At what age, or in what year, were you first told you were clinically depressed?

© 2025 Kendzerska T et al. *JAMA Network Open.*

Variables	Details
	– Are you currently taking medication for depression? (including details on the specific medications and changes in medications since the baseline)<br>– Are you currently undergoing other treatment for depression?<br>Standardized questionnaires:<br>– Depression was also measured by the Center for Epidemiologic Studies Short Depression Scale (CES-D).[10,11] The CES-D10 includes 10 items comprising six scales reflecting major facets of depression: depressed mood, feelings of guilt and worthlessness, feelings of helplessness and hopelessness, psychomotor retardation, loss of appetite, and sleep disturbance. Response options range from 1 to 4 for each item (1: All of the time (5-7days); 2: Occasionally (3-4 days); 3: Some of the time (1-2 days); 4: Rarely or never (less than 1 day)). The CESD-10 produces a continuous score with high scores indicating greater depressive symptoms; the cutoff score of 10 points (equivalent to the full CESD of 16 points) identified individuals with clinically relevant symptoms of depression.[12] Reliability statistics with the 10-item CES-D were found to be comparable to those reported for the original full CESD; the sensitivity of the 10-item CES-D was 97%; specificity, 84%; and positive predictive value, 85%.[13]<br>– Psychological distress will be assessed using Kessler's Psychological Distress Scale (K10).[14] This commonly-used 10-item scale has high levels of internal consistency (Cronbach's alpha = 0.88) and convergent validity (0.84).[14,15] K10 screens for symptoms of psychological distress, such as depression and anxiety, within the past month.[15] Scores were on a K10 range between 10 and 50, with scores under 20 indicating good mental health, 20–24 indicating mild mental disorder, 25–29 indicating moderate mental disorder, and 30 and above indicating the likelihood of a severe mental disorder.[14,15] The cutoff score for psychological distress recommended by Kessler is a score greater than or equal to 20.[14]
**CLSA covariates** (collected at baseline and follow-up)	1. ***Demographic and lifestyle measures*** from the CLSA database utilized previously:[4] Age, sex, gender identity, sexual orientation, education, total household income, years of education, language, perceived social standing, race/ethnicity* (White, Chinese, South Asian, Black, Filipino, Latin American, Southeast Asian, Arab, West Asian, Japanese, Korean, Other, Don't know, Refused, Other, Specify), satisfaction with income, physical activity, smoking habits, alcohol consumption, and diet were assessed on a self-report basis and will be included in our analyses. Height and weight were measured at the time of the interview and will be used to calculate the body mass index (BMI). Gender identity was introduced in Follow 1 cohort as: "By gender identity, we mean the inner sense that you have of yourself as being male or female. Gender identity can be different from your identified sex at birth or your sexual orientation, and it can change over time: Male, Female, Transgender Man/Transman, Transgender Woman/Transwoman, Genderqueer, Other."<br>2. ***Medical conditions***: Participants self-reported at baseline and follow-up whether a doctor had ever told them that they had any of a range of chronic conditions, including hypertension, cardiovascular conditions, parkinsonism, diabetes, hypothyroidism, ischemic heart disease, chronic airflow obstruction, neurological disorders, cancer, and chronic pain. Self-reported, clinician-diagnosed, chronic conditions have been shown to have high test-retest reliability in population-based health surveys.[16,17] Definition for chronic conditions: "Now I'd like to ask about any chronic health conditions which you may have." We are interested in "long-

Variables	Details
	term conditions" that are expected to last, or have already lasted, 6 months or more and have been diagnosed by a health professional.
**Cardiometabolic health** (collected at baseline)	Relevant questions:<br>– Has a doctor ever told you that you have heart disease (including congestive heart failure, or CHF)?<br>– Has a doctor ever told you that you have peripheral vascular disease or poor circulation in your limbs?<br>– IHD: Has a doctor ever told you that you have angina (or chest pain due to heart disease)? Has a doctor ever told you that you have had a heart attack or myocardial infarction? Have you ever had coronary artery bypass surgery, angioplasty, stent, or balloon angioplasty for your heart disease? Has a doctor ever told you that you have a blockage in your arteries? Are you currently taking any medications for heart disease?<br>– Has a doctor ever told you that you have diabetes, borderline diabetes or that your blood sugar is high? Are you currently taking medication for diabetes?<br>– Has a doctor ever told you that you have high blood pressure or hypertension? Are you currently taking medications for high blood pressure or hypertension?<br>– Has a doctor ever told you that you have experienced a Stroke or CVA? (cerebrovascular accident)? Are you currently taking medications for stroke?<br>***Cardiometabolic morbidity*** was identified through self-reported diagnoses (hypertension, diabetes, heart disease, peripheral vascular disease, angina, cerebrovascular accident) and relevant medications. Additionally, hypertension will be described as systolic blood pressure ≥140 or diastolic blood pressure ≥90 measured at the time of interview (mean of four measures) or self-reported history of being diagnosed with hypertension/taking antihypertensive medications.

CES-D, Center for Epidemiologic Studies Short Depression Scale; CLSA, Canadian Longitudinal Study on Aging; K10, Kessler's Psychological Distress Scale; OSA, obstructive sleep apnea

* Race and ethnicity were self-identified by participants in response to the question: "People from Canada come from many different cultural and racial backgrounds. Are you…?" (CLSA Data Dictionary). Response options were defined by the CLSA investigators and selected by the participant from a standardized list, including Arab, Black, Chinese, Filipino, Japanese, Korean, Latin American, South Asian, Southeast Asian, West Asian, White, Other (with specification), Don't know, and Refused. Canadian Longitudinal Study on Aging (CLSA). CLSA Data Dictionary – Comprehensive Cohort, Baseline. Hamilton, ON: McMaster University; available at: www.clsa-elcv.ca/data-access. The CLSA collects information on race and ethnicity as part of its core sociodemographic profile to understand diversity in aging experiences across Canada.[18] These data allow researchers to examine how social, cultural, and structural factors, including discrimination and inequities linked to race and ethnicity, may influence health trajectories, access to care, and determinants of healthy aging.

© 2025 Kendzerska T et al. *JAMA Network Open.*

eTable 2. Flow of Participants and Derivation of Final Analytic Samples Across Study Analyses

Analytic approach used		N missing	Sample size (N)	
**A cross-sectional analysis: Baseline Model**			30,097	**Total sample size of original data***
	Missing data on high risk for OSA	2,826	27,271	
	Missing data on composite mental health outcome	321	26,950	**Univariate sample size**
	Missing other covariates in the final model	3,485	23,465	**Multivariate sample size**
**A cross-sectional analysis: Follow-up Model**			27,765	**Total sample size of original data**
	Missing data on high risk for OSA	2,744	25,021	
	Missing data on composite mental health outcome	430	24,591	**Univariate sample size**
	Missing data on other covariates in the final model	3,351	21,240	**Multivariate sample size**
**A longitudinal analysis on Incident Cohort**			30,097	**Total sample size of original data**
	No composite mental health outcome at baseline		18,622	**Incident cohort**
	Missing data on high risk for OSA	897	17,725	
	Missing data on composite mental health outcome	1,389	16,336	**Univariate sample size**
	Missing data on other covariates in the final model	1,851	14,485	**Multivariate sample size**
**Mixed Regression Model**	**Among 23,465 at baseline and 21,240 at follow-up, 16,210 participants had info had at both time points**			
	**Therefore, the sample size is 2*16,210=32,420**			

* Among the 51,338 CLSA participants recruited at baseline (www.clsa-elcv.ca/), data were collected from 21,241 participants through telephone interviews. This group is referred to as the Tracking cohort. The remaining 30,097 participants provide data through in-home interviews and Data Collection Site visits. This group is referred to as the Comprehensive cohort, which was used in our study.

© 2025 Kendzerska T et al. *JAMA Network Open.*

eTable 3. Population Characteristics of Variables Considered in the Statistical Model at Baseline and Follow-up

Variables	Baseline, N (%)	High OSA risk, N (%)		P value	Follow-up, N (%)	High OSA risk, N (%)		P value
	**Total**	**Yes**	**No**		**Total**	**Yes**	**No**	
	**N=30,097**	**N = 7,066**	**N = 20,205**		**N=27,765**	**N=7,493**	**N= 17,528**	
**Start Date Year**				<0.001				0.01
2011-2012 (combined to avoid cells <5)	3,803 (12.63)	842 (11.92)	2,572 (12.73)					
2013	11,476 (38.13)	2,516 (35.61)	7,803 (38.62)					
2014	10,363 (34.43)	2,457 (34.77)	6,993 (34.61)					
2015	4,455 (14.8)	1,251 (17.70)	2,837 (14.04)		4,328 (15.59)	1,159 (15.47)	2,719 (15.51)	
2016					10,103 (36.39)	2,616 (34.91)	6,548 (37.36)	
2017					10,656 (38.38)	2,963 (39.54)	6,644 (37.91)	
2018					2,678 (9.65)	755 (10.08)	1,617 (9.23)	
**Missing***		2,826				2,744		
**Sociodemographic and lifestyle measures**								
**Cultural / Racial Background**				0.48				
White only	28,372 (94.27)	6,656 (94.29)	19,101 (94.63)					
Black only	221 (0.73)	53 (0.75)	139 (0.69)					
Korean only or West Asian only (combined to avoid cells <5)	42 (0.14)	7 (0.10)	25 (0.12)					
Filipino only	47 (0.16)	9 (0.13)	30 (0.15)					
Japanese only	37 (0.12)	9 (0.13)	23 (0.11)					
Chinese only	212 (0.70)	42 (0.59)	138 (0.68)					
South Asian only	271 (0.90)	69 (0.98)	176 (0.87)					
Southeast Asian only	52 (0.17)	9 (0.13)	30 (0.15)					
Arab only	89 (0.30)	13 (0.18)	67 (0.33)					
Latin American only	103 (0.34)	26 (0.37)	60 (0.30)					
Other racial or cultural origin (only)	174 (0.58)	47 (0.67)	105 (0.52)					
Multiple racial or cultural origins	447 (1.49)	119 (1.69)	291 (1.44)					
Missing	30 (0.10)							
**Missing***		2,853						
**Rural status: Yes**	2,424 (8.05)	572 (8.10)	1,633 (8.08)	0.97				
**Missing***		2,826						

© 2025 Kendzerska T et al. *JAMA Network Open.*

Variables	Baseline, N (%)	High OSA risk, N (%)		P value	Follow-up, N (%)	High OSA risk, N (%)		P value
	Total	Yes	No		Total	Yes	No	
	N=30,097	N = 7,066	N = 20,205		N=27,765	N=7,493	N= 17,528	
**Sexual orientation**								
Heterosexual	2,6637 (97.8)	6,909 (97.94)	19728 (97.76)	0.33				
Homosexual	486 (1.78)	113 (1.60)	373 (1.85)					
Bisexual	112 (0.41)	32 (0.45)	80 (0.40)					
**Missing***		2,862						
**Age groups**				<0.001				
45-54	7,595 (25.24)	1,439 (20.37)	5,646 (27.94)		4,394 (15.83)	988 (13.19)	3,149 (17.97)	<0.001
55-64	9,856 (32.75)	2,486 (35.18)	6,595 (32.64)		9,173 (33.04)	2,522 (33.66)	6,014 (34.31)	
65-74	7,362 (24.46)	1,940 (27.46)	4,675 (23.14)		8,243 (29.69)	2,506 (33.44)	4,917 (28.05)	
75+	5,284 (17.56)	1,201 (17)	3,289 (16.28)		5,955 (21.45)	1,477 (19.71)	3,448 (19.67)	
**Missing***		2,826				2,744		
**Sex: Female**	15,320 (50.9)	2,688 (38.04)	11,043 (54.65)	<0.001	14,133 (50.9)	2,889 (38.56)	9,695 (55.31)	<0.001
**Missing**		2,826				2,744		
**Married?**				0.81				0.31
Missing	8 (0.03)				11 (0.04)			
Yes	20,651 (68.61)	5,022 (71.09)	14,330 (70.94)		18,855 (67.91)	5,308 (70.86)	12,303 (70.22)	
No	9,438 (31.36)	2,042 (28.91)	5,869 (29.06)		8,899 (32.05)	2,183 (29.14)	5,218 (29.78)	
**Missing***		2,834				2,753		
**Dwelling Type**				<0.001				0.01
Missing	10 (0.03)				1 (0)			
House	24,004 (79.76)	5,634 (79.77)	16,462 (81.5)		21,671 (78.05)	5,878 (78.46)	14,001 (79.88)	
Apartment or condominium	5,810 (19.3)	1,347 (19.07)	3,590 (17.77)		5,474 (19.72)	1,456 (19.43)	3,201 (18.26)	
Other	273 (0.91)	Not reported to avoid cells <5			619 (2.23)	46 (0.61)	89 (0.51)	
**Missing**		2,836				2,745		
**Total household income**				<0.001				<0.001
Missing	1,941 (6.45)				1,771 (6.38)			
< $50,000	7,926 (26.33)	2,004 (30.2)	4,682 (24.71)		6,879 (24.78)	1,942 (27.56)	3,847 (23.34)	
≥ $50,000 and < $100,000	9,907 (32.92)	2,348 (35.39)	6,783 (35.8)		9,406 (33.88)	2,603 (36.94)	5,964 (36.19)	
≥ $100,000 and < $150,000	5,524 (18.35)	1,282 (19.32)	3,897 (20.57)		5,124 (18.45)	1,390 (19.72)	3,411 (20.7)	
$150,000 or more	4,799 (15.95)	1,001 (15.09)	3,584 (18.92)		4,585 (16.51)	1,112 (15.78)	3,258 (19.77)	
**Missing***		4,516				4,238		

© 2025 Kendzerska T et al. *JAMA Network Open.*

Variables	Baseline, N (%)	High OSA risk, N (%)		P value	Follow-up, N (%)	High OSA risk, N (%)		P value
	Total	Yes	No		Total	Yes	No	
	N=30,097	N = 7,066	N = 20,205		N=27,765	N=7,493	N= 17,528	
**Education high school (at baseline only): Yes**	26,847 (89.2)	6,124 (94.52)	18,386 (96.45)	<0.001				
Missing	2054 (6.82)							
**Missing***		4,556						
**Satisfaction with Life Scale (SWLS) - Revised**								
Missing	354 (1.18)			<0.001	356 (1.28)			<0.001
Dissatisfied/Neutral	4,126 (13.71)	1,225 (17.5)	2,308 (11.55)		3,470 (12.5)	1,176 (15.9)	1,782 (10.28)	
Slightly satisfied	4,471 (14.86)	1,205 (17.21)	2,803 (14.03)		3,441 (12.39)	1,047 (14.16)	1,986 (11.45)	
Satisfied	9,221 (30.64)	2,175 (31.07)	6,220 (31.13)		8,222 (29.61)	2,241 (30.3)	5,163 (29.78)	
Extremely satisfied	11,925 (39.62)	2,395 (34.21)	8,652 (43.3)		12,276 (44.21)	2,931 (39.63)	8,407 (48.49)	
**Missing***		3,114				3,032		
**Self-rated general health**				<0.001				<0.001
Missing	23 (0.08)				38 (0.14)			
Excellent	5,995 (19.92)	765 (10.84)	4,834 (23.94)		5,071 (18.26)	911 (12.18)	3,791 (21.65)	
Very good	12,420 (41.27)	2,518 (35.67)	8,869 (43.93)		11,460 (41.27)	2,704 (36.15)	7,798 (44.52)	
Good	8,877 (29.49)	2,612 (37)	5,260 (26.05)		8,199 (29.53)	2,661 (35.58)	4,631 (26.44)	
Fair	2,315 (7.69)	949 (13.44)	1,046 (5.18)		2,450 (8.82)	982 (13.13)	1,082 (6.18)	
Poor	467 (1.55)	216 (3.06)	182 (0.9)		547 (1.97)	221 (2.95)	212 (1.21)	
**Missing***		2,846				2,772		
**Functional Social Support**				<0.001				<0.001
Missing	606	140	336		1,356	210	399	
Median (IQR)	85.5 (71.1-96.1)	84.2 (71.1-94.7)	85.5 (73.7-96.1)		85.5 (72.4-96.1)	84.2 (71.1-96.1)	86.8 (73.7-97.4)	
**Current Smoker?**				0.16				<0.001
Missing	9,603 (31.91)				29 (0.1)			
Yes	2,576 (8.56)	642 (12.48)	1,565 (11.73)		1,944 (7)	600 (8.01)	1,088 (6.21)	
No	17,918 (59.53)	4,502 (87.52)	11,780 (88.27)		25,792 (92.89)	6,887 (91.99)	16,421 (93.79)	
**Missing***		11,608				2,769		
**Ever alcohol consumption (Yes)**	29,383 (97.63)	6,907 (97.75)	19,747 (97.74)	0.95	27,033 (97.36)	7,310 (97.56)	17,072 (97.4)	0.46
Missing	1 (0)				2 (0.01)			

© 2025 Kendzerska T et al. *JAMA Network Open.*

Variables	Baseline, N (%)	High OSA risk, N (%)		P value	Follow-up, N (%)	High OSA risk, N (%)		P value
	Total	Yes	No		Total	Yes	No	
	N=30,097	N = 7,066	N = 20,205		N=27,765	N=7,493	N= 17,528	
**Missing**		2,827				2,744		
**Type of Drinker (Past 12 Months)**				<0.001				<0.001
Missing	726 (2.41)				21 (0.08)			
Regular drinker (at least once a month)	22,239 (73.89)	5,010 (72.57)	15,385 (77.94)		20,889 (75.24)	5,522 (73.74)	13,588 (77.57)	
Occasional drinker	3,705 (12.31)	987 (14.3)	2,277 (11.53)		3,405 (12.26)	970 (12.95)	1,945 (11.1)	
Did not drink in the last 12 months	3,427 (11.39)	907 (13.14)	2,078 (10.53)		3,450 (12.43)	996 (13.3)	1,984 (11.33)	
**Missing***		3,453				2,760		
**On average, how many hours per day did you spend walking?**				<0.001				<0.001
Missing	5,731 (19.04)				4,927 (17.75)			
< 30 minutes	5,053 (16.79)	1,397 (24.53)	3,363 (19.32)		4,986 (17.96)	1,498 (25.45)	2,910 (19.62)	
30 minutes but less than 1 hour	10,633 (35.33)	2,439 (42.83)	7,691 (44.19)		10,075 (36.29)	2,531 (43)	6,675 (45.01)	
1 hour but less than 2 hours	6,707 (22.28)	1,412 (24.8)	4,941 (28.39)		5,945 (21.41)	1,416 (20.06)	4,021 (27.11)	
>=2 hours	1,973 (6.56)	446 (7.83)	1,409 (8.1)		1,832 (6.6)	441 (7.49)	1,225 (8.26)	
**Missing***		6,999				7,048		
**BMI**				<0.001				<0.001
Missing	136 (0.45)				1,020 (3.67)			
Underweight	217 (0.72)	13 (0.19)	180 (0.89)		210 (0.76)	24 (0.33)	167 (0.97)	
Normal weight	8,863 (29.45)	1,004 (14.31)	7,146 (35.48)		7,936 (28.58)	1,151 (15.63)	6,261 (36.22)	
Overweight	12,088 (40.16)	2,624 (37.41)	8,309 (41.25)		10,738 (38.67)	2,743 (37.26)	7,184 (41.56)	
Obese - Class I	5,820 (19.34)	1,929 (27.5)	3,273 (16.25)		5,162 (18.59)	2,002 (27.19)	2,658 (15.38)	
Obese - Class II	1,978 (6.57)	887 (12.65)	904 (4.49)		1,777 (6.4)	904 (12.28)	713 (4.13)	
Obese - Class III	995 (3.31)	557 (7.94)	329 (1.63)		922 (3.32)	538 (7.31)	301 (1.74)	
**Missing**		2,942				3,119		
**Medical conditions**								
**# medications taken by the participant**				<0.001				<0.001
Missing	0	0	0		3	98	190	
Median (IQR)	4 (2-7)	5 (2-8)	3 (1-6)		4 (2-7)	5 (2-8)	3 (1-6)	

© 2025 Kendzerska T et al. *JAMA Network Open.*

Variables	Baseline, N (%)	High OSA risk, N (%)		P value	Follow-up, N (%)	High OSA risk, N (%)		P value
	**Total**	**Yes**	**No**		**Total**	**Yes**	**No**	
	**N=30,097**	**N = 7,066**	**N = 20,205**		**N=27,765**	**N=7,493**	**N= 17,528**	
**TBI (Yes)**	7,288 (24.22)	2,015 (28.52)	4,641 (22.97)	<0.001	6,475 (23.32)	2,135 (28.96)	3,898 (22.55)	<0.001
Missing					1,143 (4.12)			
**Missing***		2,826				3,111		
**Usually free of pain and discomfort**				<0.001				<0.001
Missing	1,343 (4.46)				644 (2.32)			
Yes	18,130 (60.24)	3,749 (53.73)	13,517 (66.97)		18,208 (65.58)	4,444 (59.4)	12,435 (71.06)	
No	10,624 (35.3)	3,228 (46.27)	6,666 (33.03)		8,913 (32.1)	3,038 (40.6)	5,064 (28.94)	
**Missing***		2,937				2784		
**Usual intensity of pain or discomfort**				<0.001				<0.001
Missing	19,635 (65.24)				18,920 (68.14)			
Mild	4,589 (15.25)	1,243 (39.04)	3,075 (46.75)		3,864 (13.92)	1,208 (40.09)	2,391 (47.47)	
Moderate	4,901 (16.28)	1,547 (48.59)	3,007 (45.71)		4,119 (14.84)	1,439 (47.76)	2,261 (44.89)	
Severe	972 (3.23)	394 (12.37)	496 (7.54)		862 (3.1)	366 (12.15)	385 (7.64)	
**Missing**		20,335				19,715		
**Diabetes (Yes)**	5,310 (17.64)	2,025 (28.74)	2,674 (13.27)	<0.001	5,307 (19.11)	2,227 (30.1)	2,599 (14.99)	<0.001
Missing	110 (0.37)				1,050 (3.78)			
**Missing**		2,907				3,024		
**Respiratory Problem? (Yes)**	7,990 (26.55)	2,347 (33.46)	4,787 (23.82)	<0.001	7,337 (26.43)	2,548 (34.52)	4,175 (24.16)	<0.001
Missing	200 (0.66)				1,146 (4.13)			
**Missing**		2,983				3,100		
**Hypertension (Yes)**	14,127 (46.94)	5,777 (82.11)	6,601 (32.87)	<0.001	13,714 (49.39)	6,273 (84.95)	6,088 (35.58)	<0.001
Missing	268 (0.89)				1,509 (5.43)			
**Missing**		2,976				3,269		
**CVD / stroke (Yes)**	3,301 (10.97)	1,182 (16.85)	1,667 (8.29)	<0.001	3,236 (11.65)	1,293 (17.54)	1,581 (9.15)	0.10
Missing	209 (0.69)				1,149 (4.14)			
**Missing**		2,984				3,113		
**Ever cancer told by doctor (Yes)**	4,637 (15.41)	1,164 (16.51)	2,952 (14.65)	<0.001	4,972 (17.91)	1,444 (19.5)	3,073 (17.72)	0.0009
Missing	93 (0.31)				1,036 (3.73)			
**Missing**		2,893				3,013		

© 2025 Kendzerska T et al. *JAMA Network Open.*

Variables	Baseline, N (%)	High OSA risk, N (%)		P value	Follow-up, N (%)	High OSA risk, N (%)		P value
	**Total**	**Yes**	**No**		**Total**	**Yes**	**No**	
	**N=30,097**	**N = 7,066**	**N = 20,205**		**N=27,765**	**N=7,493**	**N= 17,528**	
**Under-active thyroid gland (Yes)**	3,962 (13.16)	949 (13.58)	2583 (12.93)	0.16	3,989 (14.37)	1065 14.48	2557 14.84	0.48
Missing	394 (1.31)				1,230 (4.43)			
**Missing**		3,131				3,177		
**Sleep complaints and other sleep disorders aside from OSA**								
**Number of sleep hours during the past month**				<0.001				<0.001
Missing	69	26	33		909	28	51	
Median (IQR)	7 (6-8)	7 (6-8)	7 (6-8)		7 (6-8)	7 (6-8)	7 (6-8)	
**Having symptoms of sleep-onset insomnia (Yes)**	4,740 (15.75)	1,268 (17.99)	2,930 (14.53)	<0.001	4,228 (15.23)	1,308 (17.54)	2,544 (14.56)	<0.001
Missing	68 (0.23)				936 (3.37)			
**Missing**		2,884				2,834		
**Having symptoms of sleep-maintenance insomnia (Yes)**	7,194 (23.9)	1,996 (28.3)	4,488 (22.33)	<0.001	6,531 (23.52)	2,060 (27.58)	3,969 (22.7)	<0.001
Missing	35 (0.12)				895 (3.22)			
**Missing**		2,851				2,810		
**Insomnia (difficulty falling asleep or maintaining sleep and functional impairment): Yes**	1,299 (4.32)	564 (8.02)	608 (3.02)	<0.001	992 (3.57)	458 (6.17)	473 (2.72)	<0.001
Missing	133 (0.44)				1,056 (3.8)			
**Missing**		2,928				2,923		
**Restless Leg Syndrome (Yes)**	7,809 (25.95)	2,272 (32.25)	4,788 (23.75)	<0.001	5,032 (18.12)	1,704 (22.85)	2,920 (16.73)	<0.001
Missing	72 (0.24)				955 (3.44)			
**Missing**		2,893				2,855		
**Acts out on dreams while asleep (Yes)**	3,328 (11.05)	1,096 (15.68)	1,921 (9.58)	<0.001	2,859 (10.3)	1,121 (15.31)	1,563 (90.98)	<0.001
Missing	277 (0.92)				1,333 (4.8)			

© 2025 Kendzerska T et al. *JAMA Network Open.*

Variables	Baseline, N (%)	High OSA risk, N (%)		P value	Follow-up, N (%)	High OSA risk, N (%)		P value
	**Total**	**Yes**	**No**		**Total**	**Yes**	**No**	
	**N=30,097**	**N = 7,066**	**N = 20,205**		**N=27,765**	**N=7,493**	**N= 17,528**	
**Missing**		3,046				3,118		
**Overall dissatisfaction with sleep pattern**			<0.0001					<0.001
Missing	24 (0.08)				847 (3.05)			
Yes	7,728 (25.68)	2,309 (32.71)	4,665 (23.1)			2,226 (29.75)	3,974 (22.7)	
No	22,345 (74.24)	4,750 (67.29)	15,530 (76.9)		20,240 (72.9)	5,257 (70.25)	13,530 (77.3)	
**Missing**		2,843				2,778		

BMI, body mass index; CVD, cardiovascular conditions; OSA, obstructive sleep apnea; TBI, traumatic brain injury

* the difference in the total and by OSA status is explained by the missing values by the OSA status.

Estimates are presented as frequencies and percentages, unless otherwise specified.

The final list of variables at baseline (N=19): age, sex, dwelling type, total household income, self-rated general health, satisfaction with life, alcohol consumption, BMI, self-reported hypertension, diabetes, respiratory problem, usual intensity of pain or discomfort, positive screen for traumatic brain injury, under-active thyroid gland, the number of medications taken, self-reported acting out on dream, restless legs, number of sleep hours per night, insomnia with daytime impairment.

The final list of variables at follow-up (N=17): age, sex, dwelling type, total household income, satisfaction with life, current smoker status, alcohol consumption, self-reported cardiovascular and cerebrovascular conditions, diabetes, respiratory problem, usual intensity of pain or discomfort, positive screen for traumatic brain injury, under-active thyroid gland, self-reported acting out on dream, restless legs, number of sleep hours per night, insomnia with daytime impairment.

© 2025 Kendzerska T et al. *JAMA Network Open.*

eTable 4. The Distribution of the Composite Poor Mental Health Outcome and its Components at Baseline and Follow-up

Outcomes		Baseline (N=30,097)		Follow-up (N=27,765)	
		N	%	N	%
**Anxiety disorder**	Missing	121	0.4	1,061	3.82
based on self-reported physician diagnoses of an anxiety disorder "such as a phobia, obsessive-compulsive disorder or a panic disorder"	yes	2,597	8.63	2,558	9.21
**Mood disorder**	Missing	109	0.36	1,063	3.83
based on self-reported physician diagnoses of a mood disorder "such as depression (including manic depression), bipolar disorder, mania, or dysthymia"	yes	5,144	17.09	4,980	17.94
**Clinical Depression**	Missing	152	0.51	1,177	4.24
based on self-reported physician diagnoses of a "clinical depression"	yes	4,919	16.34	3,931	14.16
**Screen for depression** based on the Center for Epidemiologic Studies Short Depression Scale (CESD)-10≥10	Missing	126	0.42	1,104	3.98
	Positive	4,768	15.84	3,750	13.51
**Psychological Distress** based on Kessler Psychological Distress Scale (K10)≥20	Missing	1679	5.58	297	1.07
	yes	3,283	10.91	2,699	9.72
**Self-reported antidepressant use** ("are you currently taking medications for depression?")	Missing	25247	83.89	23847	85.89
	yes	2,416	8.03	1,874	6.75
	no	2,434	8.09	2,044	7.36
**Composite poor mental health outcome**	Missing	1141	3.79	1132	4.08
	Yes	10,334	34.34	8,851	31.88

© 2025 Kendzerska T et al. *JAMA Network Open.*

eTable 5. The Association Between Exposures and Changes in the Center for Epidemiologic Studies Short Depression Scale (CESD-10) or Kessler Psychological Distress Scale (K10) Over Time, Considered Separately as Continuous Variables. Estimates and 95% confidence interval (CI) are presented from multivariable mixed-effects linear regressions.

	**Estimate**	**Lower CI**	**Upper CI**	**P value**
**High risk of OSA: Yes vs. No**				
CESD-10 (Center for Epidemiologic Studies Depression Scale - 10 items)	0.57	0.46	0.67	< 0.0001
K10 (Kessler Psychological Distress Scale)	0.53	0.43	0.64	< 0.0001
**Stopped breathing in sleep: Yes vs. No**				
CESD-10 (Center for Epidemiologic Studies Depression Scale - 10 items)	0.31	0.20	0.43	< 0.0001
K10 (Kessler Psychological Distress Scale)	0.38	0.26	0.50	< 0.0001

© 2025 Kendzerska T et al. *JAMA Network Open.*

eTable 6. The Association Between the High Obstructive Sleep Apnea (OSA) Risk and the Composite Poor Mental Health Outcome in the Fully Adjusted Statistical Model With and Without the Interaction Terms. Estimates are presented as odds ratios and 95% confidence intervals.

	**Cross-sectional associations**		**Longitudinal associations**	**Repeated measures analysis**
	**Baseline**	**Follow-up**	**Free from the composite outcome at baseline**	**Mixed regression**
**Full model with no interaction terms***				
High risk for OSA: Yes vs. No	1.30 (1.17-1.45)	1.30 (1.18-1.43)	1.18 (0.96-1.45)	1.11 (1.02-1.20)
**Full model with interaction term between age and high OSA risk**				
↑ in age by 1 year (high risk OSA = Yes)	0.97 (0.96-0.98)	0.97 (0.96-0.97)	1.00 (0.98-1.02)	0.97 (0.96-0.98)
↑ in age by 1 year (high risk OSA = No)	0.97 (0.97-0.98)	0.97 (0.96-0.97)	0.99 (0.98-1.00)	0.97 (0.97-0.98)
High risk for OSA at age 65 years old (the median at baseline): Yes vs. No	1.28 (1.15-1.43)	1.29 (1.17-1.43)	1.20 (0.97-1.47)	1.10 (1.01-1.20)
interaction term P value	0.31	0.57	0.29	0.58
**Full model with interaction term between sex and high OSA risk**				
Male vs. Female (high risk OSA = Yes)	0.53 (0.45-0.63)	0.56 (0.51-0.62)	0.54 (0.39-0.75)	0.52 (0.45-0.60)
Male vs. Female (high risk OSA = No)	0.62 (0.56-0.69)	1.07 (0.97-1.19)	0.59 (0.49-0.72)	0.59 (0.53-0.65)
High risk for OSA (Male): Yes vs No	1.22 (1.07-1.40)	1.24 (1.10-1.40)	1.14 (0.88-1.46)	1.05 (0.95-1.16)
High risk for OSA (Female): Yes vs No	1.43 (1.22-1.68)	1.37 (1.20-1.57)	1.25 (0.92-1.69)	1.20 (1.06-1.35)
Interaction term P value	0.12	0.25	0.62	0.09

© 2025 Kendzerska T et al. *JAMA Network Open.*

eTable 7. Characteristics of Individuals With High Obstructive Sleep Apnea (OSA) Risk Associated With New Mental Health Conditions. Estimates (odds ratios [OR] and 95% confidence intervals [CI]) are presented from (i) conventional multivariable logistic regression (in individuals with high OSA risk without concurrent mental health conditions at baseline), and (ii) mixed multivariable logistic regression (in individuals with high OSA risk regardless of mental health status).

**Characteristics**	**Population: high OSA risk without concurrent mental health conditions at baseline**				**Population: high OSA risk regardless of mental health status at baseline**			
	**odds ratio**	**LCI**	**UCI**	**P value**	**odds ratio**	**LCI**	**UCI**	**P value**
Age, years (increase in one year)	1.00	0.99	1.02	0.5109	0.97	0.96	0.97	< 0.001
Male vs. Female	0.65	0.50	0.84	0.0011	0.60	0.54	0.66	< 0.001
Dwelling Type								
Apartment vs. House	1.15	0.84	1.56	0.383	1.31	1.14	1.50	< 0.001
Other vs. House	1.53	0.42	5.63	0.5208	1.21	0.80	1.82	0.36
Total household income								
50-100K vs. < 50K	0.73	0.55	0.99	0.0418	0.92	0.81	1.05	0.23
100-150K vs. < 50K	0.76	0.53	1.09	0.1323	0.72	0.62	0.85	< 0.001
150K+ vs. < 50K	0.71	0.47	1.07	0.0997	0.77	0.64	0.91	0.00
Self-rated general health								
Very Good vs. Excellent	1.01	0.69	1.48	0.9674	1.35	1.14	1.59	0.00
Good vs. Excellent	1.26	0.85	1.87	0.2518	1.61	1.35	1.92	< 0.001
Fair vs. Excellent	1.90	1.13	3.17	0.0148	2.36	1.87	2.97	< 0.001
Poor vs. Excellent	1.11	0.38	3.25	0.8502	2.18	1.44	3.29	0.00
Satisfaction with Life								
Slightly satisfied vs. Dissatisfied/Neutral	0.74	0.48	1.12	0.1533	0.55	0.46	0.66	< 0.001
Satisfied vs. Dissatisfied/Neutral	0.65	0.44	0.95	0.0258	0.33	0.28	0.39	< 0.001
Extremely satisfied vs. Dissatisfied/Neutral	0.48	0.32	0.70	0.0002	0.25	0.21	0.29	< 0.001
Alcohol Frequency								
>2 times a week vs. None	1.06	0.71	1.59	0.7591	0.85	0.71	1.01	0.07
Once a week vs. None	0.78	0.50	1.20	0.2562	0.70	0.58	0.85	0.00
Once a month vs. None	1.03	0.67	1.58	0.9103	0.91	0.75	1.10	0.32
Body mass index (increase in one unit of BMI)	1.00	0.98	1.02	0.9464	0.99	0.98	1.00	0.02
Comorbidities: Yes vs. No								
Diabetes	1.11	0.85	1.46	0.4502	1.08	0.96	1.23	0.20
Respiratory Problem	1.19	0.93	1.53	0.1713	1.28	1.15	1.43	< 0.001
Pain intensity: Mild vs. None	1.09	0.81	1.48	0.5701	1.43	1.24	1.64	< 0.001
Pain intensity: Moderate vs. None	0.91	0.66	1.26	0.5702	1.74	1.52	1.99	< 0.001

© 2025 Kendzerska T et al. *JAMA Network Open.*

Characteristics	Population: high OSA risk without concurrent mental health conditions at baseline				Population: high OSA risk regardless of mental health status at baseline			
	**odds ratio**	**LCI**	**UCI**	**P value**	**odds ratio**	**LCI**	**UCI**	**P value**
Pain intensity: Severe vs. None	1.27	0.70	2.32	0.4307	2.17	1.65	2.85	< 0.001
Positive screen for traumatic brain injury	1.05	0.81	1.36	0.7191	1.30	1.16	1.46	< 0.001
Underactive thyroid gland	1.12	0.79	1.58	0.5256	1.02	0.88	1.19	0.76
Number of medications taken (increase by one)	1.03	1.00	1.06	0.0663	1.06	1.05	1.08	< 0.001
Other than OSA sleep-related conditions: Yes vs. No								
Acts out on a dream	1.42	1.04	1.93	0.0282	1.43	1.23	1.66	< 0.001
Restless legs	1.47	1.14	1.89	0.0026	1.31	1.17	1.47	< 0.001
Number of sleep hours per night (increase by one hour)	0.92	0.83	1.01	0.0949	1.02	0.98	1.06	0.41
Insomnia (difficulty falling asleep or maintaining sleep associated with functional impairment	2.01	1.18	3.42	0.0102	3.41	2.63	4.43	< 0.001

© 2025 Kendzerska T et al. *JAMA Network Open.*

eTable 8. Comparison of the Population Characteristics at Baseline of Individuals Excluded at the Follow-up (ie, Data Available at Baseline Only) and Individuals With Available Data at Both Time Points (Baseline and Follow-up)

Variables	Both Timepoints (N Total = 27,765)	Only Baseline (N Total = 2,332)	P value	Frequency Missing
	N (% row)	N (% row)		N total
**Exposure**				
High risk for OSA: Yes	6,625 (25.56)	441 (32.74)	<0.001	2,826
Stopped breathing in sleep: Yes	4,030 (15.00)	215 (16.03)	0.30	1,898
**Outcomes**				
Composite Mental Health	12,143(44.51)	1220 (65.77)	<0.001	959
Anxiety disorder	2,341 (8.46)	256 (11.05)	<0.001	121
Mood disorder	4,686 (16.93)	458 (19.77)	<0.001	109
Clinical Depression	4,488 (16.24)	431 (18.71)	0.002	152
Taking medication for depression	2,195 (49.6)	221 (52)	0.35	25,247
Center for Epidemiological Studies Short Depression Scale (CES-D 10): Positive screen for depression: Yes	4,421 (15.25)	547 (23.78)	<0.001	126
Center for Epidemiological Studies Short Depression Scale (CES-D 10) score, mean (SD)	5.2 (4.6)	6.5 (5.3)	<0.001	161
K10 Psychological Distress: Yes	3,025 (11.17)	258 (19.23)	<0.001	1,679
Psychological Distress Scale (K10), mean, SD	14.2 (4.5)	15.4 (5.9)	<0.001	
**Covariates**				
**Start Date Year**				
2011-2012	3,503 (12.61)	300 (12.86)	0.10	—
2013	10,606 (38.2)	870 (37.31)		
2014	9,590 (34.54)	773 (33.15)		
2015	4,066 (14.64)	389 (16.68)		
**Sociodemographic and lifestyle measures**				
**Age groups**				
45-54	7,145 (25.73)	450 (19.3)	<0.001	—
55-64	9,276 (33.41)	580 (24.87)		
65-74	6.781 (24.42)	581 (24.91)		
75+	4,563 (16.43)	721 (30.92)		
Age, years. Mean (SD)	62.7 (10.1)	66.4 (11.2)		
**Sex: Female**	14,133 (50.9)	1,187 (50.9)	0.99	—
**Cultural / Racial Background: While only**	26,240 (94.59)	2,132 (91.62)	<0.001	30
**Rural status: Yes**	2,250 (8.10)	174 (7.46)	0.27	—
**Dwelling Type**				
House	22,423 (80.79)	1,581 (67.8)	<0.001	10
Apartment/condo	5,106 (18.4)	704 (30.19)		
Other	226 (0.81)	47 (2.02)		
**Household income**				
< $50,000	6,920 (26.57)	1,006 (47.57)	<0.001	1,941
≥ $50,000 and < $100,000	9,286 (35.66)	621 (29.36)		
≥ $100,000 and < $150,000	5,281 (20.04)	306 (14.47)		
$150,000 or more	4,617 (17.73)	182 (8.61)		

© 2025 Kendzerska T et al. *JAMA Network Open.*

Variables	Both Timepoints (N Total = 27,765)	Only Baseline (N Total = 2,332)	P value	Frequency Missing
	N (% row)	N (% row)		N total
**Life Satisfaction**				
Dissatisfied/Neutral	3,616 (13.17)	510 (22.3)	<0.001	354
Slightly satisfied	4,090 (14.9)	381 (16.66)		
Satisfied	8,563 (31.19)	658 (28.77)		
Extremely satisfied	11,187 (40.75)	738 (32.27)		
**Self-rated general health**				
Excellent	5,705 (20.56)	290 (12.46)	<0.001	23
Very good	11,659 (42.02)	761 (32.69)		
Good	8,044 (28.99)	833 (35.78)		
Fair	1,970 (7.10)	345 (14.82)		
Poor	368 (1.33)	99 (4.25)		
**Current Smoker**				
Yes	2,226 (11.84)	350 (20.59)	<0.001	9,603
No	16,568 (88.16)	1,350 (79.41)		
**Ever alcohol consumption (Yes)**	27,135 (97.73)	2,248 (96.4)	<0.001	1
**Type of Drinker (Past 12 Months)**				
Regular drinker (at least once a month)	20,804 (76.69)	1,435 (63.92)	<0.001	726
Occasional drinker	3,319 (12.24)	386 (17.19)		
Did not drink in the last 12 months	3,003 (11.07)	424 (18.89)		
**BMI**				
Underweight	181 (0.65)	36 (1.56)	<0.001	136
Normal weight	8,253 (29.84)	610 (26.44)		
Overweight	11,156 (40.34)	932 (40.4)		
Obese - Class I	5,343 (19.32)	477 (20.68)		
Obese - Class II	1,817 (6.57)	161 (6.98)		
Obese - Class III	904 (3.27)	91 (3.94)		
**Medical conditions**				
**# medications taken,** Median (IQR)	4 (1-6)	5 (2-8)	<0.001	0
**TBI (Yes)**	6,766 (24.37)	522 (22.38)	0.031	—
**Free of pain and discomfort (Yes)**	17,353 (63.42)	777 (55.9)	<0.001	1,341
**Usual intensity of pain or discomfort**				
Mild	4,402 (44.64)	187 (31.11)	<0.001	19,635
Moderate	4,580 (46.45)	321 (53.41)		
Severe	879 (8.91)	93 (15.47)		
**Diabetes (Yes)**	4,751 (17.17)	559 (24.14)	<0.001	110
**Hypertension (Yes)**	10,071 (36.74)	1,030 (44.76)	<0.001	179
**Respiratory Problem (Yes)**	7,252 (26.29)	738 (31.96)	<0.001	200
**CVD/ stroke (Yes)**	2,871 (10.41)	430 (18.7)	<0.001	209
**Underactive thyroid gland (Yes)**	4,751 (17.17)	559 (24.14)	<0.001	110
**Sleep complaints and other sleep disorders aside from OSA**				
**# of sleep hours per night,** Median (IQR)	7 (6-8)	7 (6-8)	0.07	69
**Insomnia (Yes)**	1,144 (4.14)	155 (6.69)	<0.001	133
**Restless Leg Syndrome (Yes)**	7,158 (25.84)	651 (27.95)	0.03	72

© 2025 Kendzerska T et al. *JAMA Network Open.*

**Variables**	**Both Timepoints (N Total = 27,765)**	**Only Baseline (N Total = 2,332)**	**P value**	**Frequency Missing**
	N (% row)	N (% row)		N total
**Acts out on dreams while asleep (Yes)**	3,068 (11.15)	260 (11.29)	0.84	277

BMI, body mass index; CVD, cardiovascular conditions; OSA, obstructive sleep apnea; TBI, traumatic brain injury

© 2025 Kendzerska T et al. *JAMA Network Open.*

eReferences.

1. Ambler G, Brady AR, Royston P. Simplifying a prognostic model: a simulation study based on clinical data. *Statistics in medicine*. Dec 30 2002;21(24):3803-22. doi:10.1002/sim.1422
2. Peduzzi P, Concato J, Feinstein AR, Holford TR. Importance of events per independent variable in proportional hazards regression analysis. II. Accuracy and precision of regression estimates. Randomized Controlled Trial. Research Support, U.S. Gov't, Non-P.H.S. *Journal of clinical epidemiology*. Dec 1995;48(12):1503-10.
3. Chung F, Yegneswaran B, Liao P, et al. STOP questionnaire: a tool to screen patients for obstructive sleep apnea. *Anesthesiology*. May 2008;108(5):812-21. doi:10.1097/ALN.0b013e31816d83e4
4. Zolfaghari S, Yao C, Thompson C, et al. Effects of menopause on sleep quality and sleep disorders: Canadian Longitudinal Study on Aging. *Menopause*. Mar 2020;27(3):295-304. doi:10.1097/GME.0000000000001462
5. Patel D, Tsang J, Saripella A, et al. Validation of the STOP questionnaire as a screening tool for OSA among different populations: a systematic review and meta-regression analysis. *J Clin Sleep Med*. May 1 2022;18(5):1441-1453. doi:10.5664/jcsm.9820
6. Zhao JL, Cross N, Yao CW, et al. Insomnia disorder increases the risk of subjective memory decline in middle-aged and older adults: a longitudinal analysis of the Canadian Longitudinal Study on Aging. *Sleep*. Jul 25 2022;doi:10.1093/sleep/zsac176
7. Bastien CH, Vallieres A, Morin CM. Validation of the Insomnia Severity Index as an outcome measure for insomnia research. *Sleep medicine*. Jul 2001;2(4):297-307. doi:10.1016/s1389-9457(00)00065-4
8. Buysse DJ, Reynolds CF, 3rd, Monk TH, Berman SR, Kupfer DJ. The Pittsburgh Sleep Quality Index: a new instrument for psychiatric practice and research. *Psychiatry research*. May 1989;28(2):193-213. doi:10.1016/0165-1781(89)90047-4
9. Cross NE, Carrier J, Postuma RB, et al. Association between insomnia disorder and cognitive function in middle-aged and older adults: a cross-sectional analysis of the Canadian Longitudinal Study on Aging. *Sleep*. Aug 1 2019;42(8)doi:10.1093/sleep/zsz114
10. Hann D, Winter K, Jacobsen P. Measurement of depressive symptoms in cancer patients: evaluation of the Center for Epidemiological Studies Depression Scale (CES-D). *J Psychosom Res*. May 1999;46(5):437-43. doi:10.1016/s0022-3999(99)00004-5
11. Andresen EM, Byers K, Friary J, Kosloski K, Montgomery R. Performance of the 10-item Center for Epidemiologic Studies Depression scale for caregiving research. *SAGE Open Med*. 2013;1:2050312113514576. doi:10.1177/2050312113514576
12. Andresen EM, Malmgren JA, Carter WB, Patrick DL. Screening for depression in well older adults: evaluation of a short form of the CES-D (Center for Epidemiologic Studies Depression Scale). *American journal of preventive medicine*. Mar-Apr 1994;10(2):77-84.
13. Irwin M, Artin KH, Oxman MN. Screening for depression in the older adult: criterion validity of the 10-item Center for Epidemiological Studies Depression Scale (CES-D). *Archives of internal medicine*. Aug 9-23 1999;159(15):1701-4. doi:10.1001/archinte.159.15.1701
14. Kessler RC, Barker PR, Colpe LJ, et al. Screening for serious mental illness in the general population. *Archives of general psychiatry*. Feb 2003;60(2):184-9. doi:10.1001/archpsyc.60.2.184
15. Sampasa-Kanyinga H, Zamorski MA, Colman I. The psychometric properties of the 10-item Kessler Psychological Distress Scale (K10) in Canadian military personnel. *PloS one*. 2018;13(4):e0196562. doi:10.1371/journal.pone.0196562
16. Raina PS, Wolfson C, Kirkland SA, et al. Ascertainment of chronic diseases in the Canadian longitudinal study on aging (CLSA), systematic review. *Canadian journal on aging = La revue canadienne du vieillissement*. Sep 2009;28(3):275-85. doi:10.1017/S071498080999002X
17. Raina PS, Wolfson C, Kirkland SA, et al. The Canadian longitudinal study on aging (CLSA). *Canadian journal on aging = La revue canadienne du vieillissement*. Sep 2009;28(3):221-9. doi:10.1017/S0714980809990055

© 2025 Kendzerska T et al. *JAMA Network Open.*

18. Raina P, Wolfson C, Kirkland S, et al. Cohort Profile: The Canadian Longitudinal Study on Aging (CLSA). *International journal of epidemiology*. Dec 1 2019;48(6):1752-1753j. doi:10.1093/ije/dyz173

© 2025 Kendzerska T et al. *JAMA Network Open.*
