# Supplementary material for: Obstructive Sleep Apnea Risk and Mental Health Conditions Among Older Canadian Adults in the Canadian Longitudinal Study on Aging
Source: JAMA Netw Open. 2025 Dec 26;8(12):e2549137. doi: 10.1001/jamanetworkopen.2025.49137 (PMC12743280; doi:10.1001/jamanetworkopen.2025.49137)
Supplement: Supplement 2. — Data Sharing Statement [file jamanetwopen-e2549137-s002.pdf]

Data Sharing Statement

Kendzerska. Obstructive Sleep Apnea Risk and Mental Health Conditions Among Older Canadian Adults in the Canadian Longitudinal Study on Aging. *JAMA Netw Open*. Published December 26, 2025. doi:10.1001/jamanetworkopen.2025.49137

Data
Data available: No

Additional Information
Explanation for why data not available: The Canadian Longitudinal Study on Aging (CLSA's) privacy and confidentiality requirements do not permit Approved Users to share CLSA data beyond their research team. Data are available from the Canadian Longitudinal Study on Aging (www.clsa-elcv.ca) for researchers who meet the criteria for access to de-identified CLSA data.
